# Multi-omics biomarkers of endothelial dysregulation preceding chronic lung allograft dysfunction: A prospective cohort study

**DOI:** 10.1371/journal.pmed.1004725

**Published:** 2026-06-23

**Authors:** Giulia Iacono, Christina Begka, Bailey Cardwell, Carmel Daunt, Roxanne Chatzis, Celine Pattaroni, Alana Butler, Matthew Macowan, Bronwyn Levvey, Gregory I. Snell, Glen P. Westall, Benjamin J. Marsland

**Affiliations:** 1 Department of Immunology, School of Translational Medicine, Monash University, Melbourne, Australia; 2 Lung Transplant Service, Department of Respiratory Medicine, Alfred Hospital, Melbourne, Victoria, Australia

## Abstract

**Background:**

Long-term survival of lung transplant recipients remains limited by chronic lung allograft dysfunction (CLAD). CLAD is only diagnosed following a persistent and substantial decline in lung function, after which irreversible damage to the lungs has occurred, limiting opportunities to effectively intervene at an early stage. There is a critical need for earlier detection prior to its clinical manifestation. The immunological drivers of CLAD remain unclear, limiting the development of predictive biomarkers and new therapies.

**Methods and findings:**

In this hypothesis-generating, prospective cohort study, we profiled the microbial, metabolic, lipidomic, and gene expression dynamics of longitudinally collected broncho-alveolar lavages (BALs) from 56 CLAD-free lung transplant recipients up to 30 months post-transplant, and compared BALs from 13 CLAD-free patients to BALs from 13 patients who developed CLAD. In CLAD-free patients, the first 6 months post-transplant were hallmarked by diminished microbial diversity and increased abundance of *Staphylococcus* and *Candida*, coupled with upregulated innate and adaptive immune responses, and elevated nitric oxide metabolism (FDR < 0.05). This was superseded by homeostatic tissue repair and by the reactivation of T-cell genes such as *CD3*, *GZMA*, *IL2RB*, *CD28*, *CD40LG*, and *LCK*, after tapering of maintenance immunosuppression (FDR < 0.05). In patients who developed CLAD, disease onset was preceded by the increased abundance of sphingolipids and the upregulation of glycocalyx and immune cell recruitment genes such as *HAPLN3*, *HS3ST3B1*, *SULF2*, *CHST2*, *CSGALNACT1*, *CXCR1*, *CSF3R*, *SELL*, *CXCL2*, and *CEACAM1* (FDR < 0.05), suggesting increased vascular dysfunction and immune cell graft infiltration prior to CLAD onset. Scoring against a publicly available lung single-cell dataset showed our bulk gene transcriptomics signature to be expressed by monocytes, endothelial, and T cells. In contrast to CLAD-free patients, this signature persisted after 1.5 months post-transplant and increased in intensity upon the start of lung function decline. Multi-omics integration highlighted sphingolipid molecules and genes involved in immune cell recruitment and endothelial function as candidate biomarkers associated with the onset of CLAD. This study is limited by its small sample size.

**Conclusions:**

We have identified immunological processes, metabolites, lipids, and genes associated with the onset of CLAD. Our findings are to be considered associative and not aimed at establishing causality. Future studies employing a targeted approach in independent validation cohorts, using, for example, quantitative polymerase chain reaction (PCR) and targeted mass-spectrometry, will be required to confirm these findings.

## Author summary

**Why was this study done?:**

- Patients that receive a life-saving lung transplant eventually develop chronic lung allograft dysfunction (CLAD), which causes a deterioration of their lung function and terminates in failure of the transplanted lung.
- There is a poor understanding of the mechanisms that precede the development of CLAD, resulting in no reliable molecular signals or clinical tests that can inform when CLAD will begin after a transplant, before the lungs begin to deteriorate.

**What did the researchers do and find?:**

- We monitored a cohort of 69 lung transplant recipients up to 30 months after their transplant by collecting deep lung lavages from each patient at regular time intervals. This longitudinal approach allowed us to track the trajectory of each patient post-transplant and link changes in their microbiome, gene expression, and molecular products present in the lung wash to the later development of CLAD.
- We first explored how the lungs return to health in patients that do not develop CLAD. These patients showed elevated immune responses and inflammation that subsided after 6 months in favor of steady state processes.
- Patients that developed CLAD showed increased activity of genes and molecules involved in the preservation of their lung vasculature.
- This signature was visible also in CLAD-free patients, but in contrast to CLAD-free patients, it did not resolve in CLAD patients after the first 1.5 months post-transplant, intensifying once their lung function began to decline at 12 months post-transplant.

**What do these findings mean?:**

- Our findings suggest molecular signals associated with the diagnosis of CLAD.
- This study has limitations that should be considered when interpreting the results. As this was a hypothesis-generating study in a small cohort of patients, findings only relate to a small number of patients, diagnosed with CLAD within 3 years post-transplant. In addition, because this was an observational study and lacked an independent validation group, the findings should be interpreted as associations rather than direct cause-and-effect relationships. Further research studies will be required to confirm these findings in larger lung transplantation cohorts and in patients who develop CLAD after 3 years post-transplant. After validation, key signatures may have the potential to inform patients at risk for the development of CLAD.

## Abstract 3

In a multi-omics analysis of lung fluid samples from patients who underwent lung transplantation, Giulia Iacono and colleagues explore molecular features associated with lung function decline.

## Introduction

Lung transplantation is lifesaving for patients with end-stage respiratory disease. However, their long-term survival remains hindered by the development of chronic lung allograft dysfunction (CLAD) [1]. CLAD manifests as an irreversible decline in lung function and airway obstruction in the bronchiolitis obliterans (BOS) phenotype; or interstitial fibrosis, in restrictive allograft syndrome (RAS) [2]. Known risk factors for CLAD include infection, acute rejection, and post-transplant complications, all of which increase inflammation and injury to the allograft [2]. CLAD is not diagnosed until after a 20% irreversible decline in forced expiratory volume in 1 s (FEV_1_) from baseline, potentially resulting in a missed opportunity for intervention prior to its clinical manifestation. Limited knowledge of the microbial, metabolic, and gene expression mechanisms preceding CLAD hinders the development of predictive biomarkers, which could inform swifter interventions and preventive strategies.

Molecular studies of broncho-alveolar lavage (BAL) [3–5], blood [6], and lung tissue are yielding important insights into transcriptomics processes that characterize CLAD [7,8]. Candidate CLAD biomarker genes may be related to either inflammatory pathways associated with hypoxia and angiogenesis [7], a dysregulated tissue injury response [8], or T-cell activation [9]. Single cell RNA sequencing analysis of CLAD tissue has highlighted potential roles for monocyte-derived macrophages [10], and basal cells that up-regulate MHC-I to elicit T-cell mediated cytotoxic responses [11].

Published studies on the prognostic value of airway metabolites and lipids in CLAD are still scarce. CLAD has been associated with increased taurine and pyruvate metabolism in the BAL [12], elevated sphingolipids and fatty acid metabolism [13]. How the dynamics of the wider lung metabolome and lipidome evolve prior to the onset of CLAD onset remains to be elaborated.

Using a multi-omics approach we performed microbial, transcriptomics, untargeted metabolomics and lipidomics profiling on longitudinal BALs from a total of 69 lung transplant recipients over the first 30 months post-transplant. Firstly, we focused on 56 stable CLAD-free recipients, with the aim of revealing patterns of shared homeostatic processes relevant for stable long-term lung function post-transplant. Secondly, we matched 13 CLAD-free recipients with 13 patients who developed CLAD to investigate mechanisms preceding its development and identify candidate biomarkers predictive of disease.

## Methods

### Ethics Statement

This study was approved by the Alfred Hospital Ethics Committee (ID 430/17). Formal written informed consent was provided by all patients. This study is reported as per the Strengthening the Reporting of Observational Studies in Epidemiology (STROBE) guideline (S1 STROBE Checklist). The prospective study protocol containing the analysis plan is included as a supplementary file.

### Cohort demographics and sample collection

**Fig 1:**
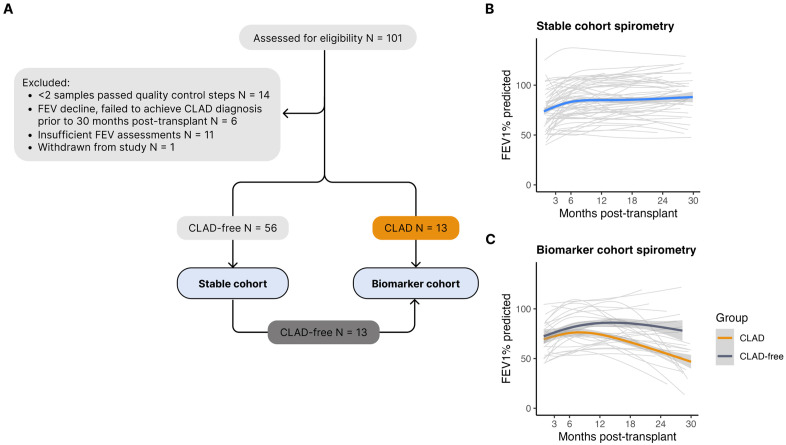
Study design. **(A)** Schematic of excluded and included patients in each study cohort. **(B, C)** Predicted forced expiratory volume 1 second percentage (FEV1%) over time in months post-transplant for the Stable (B) and Biomarker (C) cohort (linear model, normal spline with df = 2). Gray shading: 95% confidence interval of predicted FEV1%. CLAD, chronic lung allograft dysfunction.

In this prospective study, we enrolled 101 transplant recipients who underwent lung transplantation at the Alfred Hospital (Melbourne, Victoria, Australia) between March 2018 and March 2022 (Fig 1A). We excluded patients where less than 2 samples passed our quality control steps across all datasets (*N* = 14), those who developed a decline in FEV1% but did not reach a CLAD diagnosis prior to termination of the study at 30 months (*N* = 6), those with an insufficient number of FEV1% assessments (*N* = 11), and those withdrawn from the study (*N* = 1), resulting in 56 CLAD-free participants with stable lung function (Stable cohort) (Fig 1B). As a design strategy to address clinical and demographic heterogeneity in our cohort, 13 of these patients were matched to 13 patients diagnosed with CLAD prior to study termination based on age, sex, and transplant indication, for the investigation of CLAD biomarkers (Biomarker cohort) (Fig 1C). Five patients developed CLAD within 18 months (S1A Fig). Except for 2, all patients developed the BOS phenotype. At study completion, 3 of the 13 CLAD patients had CLAD grade 1, 3 had CLAD grade 2, 4 had CLAD grade 3, and 3 had CLAD grade 4. There were no differences in recipient age, time spent in ICU, transplant indication, antibiotics, azithromycin usage, positive donor-specific antibodies (DSA), clinical microbiology, primary graft dysfunction (PGD) grade, or HLA mismatch (Table 1, *p* > 0.05) (S1B Fig). Two patients experienced acute cellular rejection post-transplant, both belonging to the CLAD group (S1C Fig). Eleven patients experienced CMV reactivation in the BAL and blood (S1D Fig). For insights into biomarkers of CLAD, samples included in the Biomarker study were collected prior to CLAD (except for 4 taken within 1 month of a diagnosis). All patients were maintained on triple immunosuppression with tacrolimus, mycophenolate, corticosteroids [14]. International Society for Heart and Lung Transplantation criteria were used to define acute rejection [15] and CLAD [2]. Patients underwent surveillance bronchoscopy at 2, 6 weeks, 3, 6, 9, 12, 18 months, or if clinically indicated, with BAL sampling performed according to international guidelines [16]. Sampling was omitted in the case where patients were considered unfit for a bronchoscopy and was further delayed or restricted during the COVID-19 pandemic (March 2020–July 2021). Throughout the duration of the study, CLAD-free patients underwent 6.9 bronchoscopies on average, and 8.9 on average for CLAD patients. There was no difference in the number of samples that passed quality control steps (Table 1, samples per patient: CLAD-free: 7 (6–7); CLAD: 7 (3–9)). Details on the study design, sample collection, and processing protocols are outlined in S1 File.

**Table 1:** Biomarker cohort demographics.

	Total	CLAD-free	CLAD	pvalue
**Patients *n***	26	13	13	NA
**Recipient age at Tx (range)**	54.5 (26–70)	52.4 (26–68)	56.5 (28–70)	0.3827
**Female patients *n* (%)**	9 (35%)	6 (46%)	3 (23%)	0.2162
**Donor age at Tx (range)**	47 (15–74)	46.2 (23–74)	47.8 (15–65)	0.809
**Donor smoking *n* (%)**	17 (65%)	7 (54%)	10 (77%)	0.2162
**Ischemic time (min) (range)**	321.8 (198–539)	295.3 (198–539)	348.3 (210–504)	0.169
**Transplant type patients *n* (%)**				0.5393
BLT	23 (88%)	12 (92%)	11 (85%)	
SLT	3 (12%)	1 (8%)	2 (15%)	
**DSA at tx patients *n* (%)**	7 (27%)	4 (31%)	3 (23%)	0.6584
**Positive DSA after tx patients *n* (%)**	15 (58%)	7 (54%)	8 (62%)	0.6914
**ACR after tx patients *n* (%)**				0.6831
A2	1 (4%)	0 (%)	1 (8%)	
A1	1 (4%)	0 (%)	1 (8%)	
A0	24 (92%)	13 (100%)	11 (85%)	
**CMV mismatched patients *n* (%)**	7 (27%)	4 (31%)	3 (23%)	0.6584
**ICU hours per patient (range)**	166.9 (34–1,434)	218.3 (37–1,434)	115.5 (34–265)	0.9183
**PGD grade patients *n* (%)**				0.5543
1	11 (42%)	7 (54%)	4 (31%)	
2	10 (38%)	4 (31%)	6 (46%)	
3	4 (15%)	2 (15%)	2 (15%)	
Unknown	1 (4%)	0 (%)	1 (8%)	
**Samples *n***	172	85	87	NA
**Samples per patient (range)**	7 (3–9)	7 (6–7)	7 (3–9)	0.7657
**Average sampling days (range)**	231.5 (12–781)	235.9 (13–755)	227.3 (12–781)	0.6306
**Tx indication patients *n* (%)**				0.6049
CF/NCFB	6 (23%)	4 (31%)	2 (15%)	
CLAD	3 (12%)	1 (8%)	2 (15%)	
ILD	9 (35%)	3 (23%)	6 (46%)	
COPD	6 (23%)	3 (23%)	3 (23%)	
Other^*^	2 (8%)	2 (15%)	0 (%)	
**Induction immunosuppression patients *n* (%)**				
Basilimax	15 (58%)	7 (54%)	8 (62%)	0.6914
**Maintenance immunosuppression patients *n* (%)**				0.7478
Azathioprine	14 (54%)	6 (46%)	8 (62%)	
Cyclosporine	1 (4%)	0 (%)	1 (8%)	
Everolimus	5 (19%)	1 (8%)	4 (31%)	
Mycophenolate-Mofetil	16 (62%)	8 (62%)	8 (62%)	
Prednisolone	26 (100%)	13 (100%)	13 (100%)	
Tacrolimus	26 (100%)	13 (100%)	13 (100%)	
**Azithromycin patients *n* (%)**	16 (38%)	6 (32%)	10 (43%)	0.1069
**Antibiotics patients *n* (%)**				0.8135
Amoxicillin-clavulanate	3 (12%)	1 (8%)	2 (15%)	
Trimethoprim-sulfamethoxazole	26 (100%)	13 (100%)	13 (100%)	
Pentamidine	3 (12%)	1 (8%)	2 (15%)	
Piperacillin-Tazobactam	4 (15%)	2 (15%)	2 (15%)	
Tobramycin	4 (15%)	3 (23%)	1 (8%)	
Other^†^	6 (23%)	2 (15%)	4 (31%)	
**BAL microbiology patients *n* (%)**				0.8504
Negative	26 (60%)	13 (59%)	13 (62%)	
Positive	17 (40%)	9 (41%)	8 (38%)	

Data are number (*n*) or percentage (%) of samples or patients and mean with range (min-max). Percentages may not sum to 100% if a patient was administered multiple medications. For continuous variables: p-value calculated using the Student *t* test (if normal (Gaussian) distribution) or the Wilcoxon signed-rank test (if non-normal distribution). As determined by the Shapiro–Wilk test of normality. For categorical variables: *p*-value calculated using the chi-squared test.

Tx, transplant; BLT, bilateral lung Tx; SLT, single lung Tx; DSA, donor specific antibodies; DSA at tx is indicative of pre-formed anti-HLA DSA crossed at time of transplant; CMV, cytomegalovirus; ICU, intensive care unit; PGD, primary graft dysfunction. CF, cystic fibrosis; NCFB, non-CF bronchiectasis; CLAD, chronic lung allograft dysfunction; ILD, interstitial lung disease; COPD, chronic obstructive pulmonary disease; BAL, broncho-alveolar lavage.

* Includes patients with combined COPD/pulmonary fibrosis, hypersensitivity pneumonitis, and sarcoidosis.

† Includes Pentamidine, Trimethoprim, Meropenem, Ciprofloxacin, Vancomycin.

### 16S rRNA, ITS, RNA sequencing, and metabolomics acquisition

We performed 16S rRNA and ITS gene amplicon sequencing, metabolomics, and lipidomics (molecular features) in addition to transcriptomics on a total of 407 BALs. Detailed characteristics of samples, including the number of patients in each group and dataset, are presented in Table 2 and at the bottom of each figure legend. Bacterial and fungal DNA was isolated as previously described [17] and sequenced using Illumina MiSeq (Monash STM Genomics, Melbourne, Australia), while Illumina Novaseq6000 (Novogene, Singapore) was used for RNA sequencing. Molecular features were acquired using a QExactive mass spectrometer (Thermo Scientific) at the Monash Proteomics and Metabolomics Facility in Parkville Australia. Detailed 16S, ITS, RNA library sequencing, metabolomics extraction, acquisition are outlined in S1 File.

**Table 2:** Clinical demographics per dataset.

	Total	16S Bacteria	ITS Fungi	RNA	Small molecules
**Patients *n***	69	69	56	47	28
**Recipient age at Tx (range)**	57.2 (18–70)	57.2 (18–70)	58.2 (26–70)	56.7 (22–70)	56 (26–70)
**Female patients *n* (%)**	21 (30%)	21 (30%)	15 (27%)	17 (36%)	9 (32%)
**Donor age at Tx (range)**	48.4 (11–74)	48.4 (11–74)	49.1 (11–74)	47.4 (15–74)	44.8 (15–74)
**Donor smoking *n* (%)**	42 (61%)	42 (61%)	35 (62%)	28 (60%)	18 (64%)
**Ischemic time (min) (range)**	311.2 (105–628)	311.2 (105–628)	313.9 (141–628)	301.8 (105–628)	300.5 (155–539)
**Transplant type patients *n* (%)**					
BLT	56 (81%)	56 (81%)	47 (84%)	41 (87%)	26 (93%)
SLT	13 (19%)	13 (19%)	9 (16%)	6 (13%)	2 (7%)
**DSA at tx patients *n* (%)**	12 (17%)	12 (17%)	9 (16%)	10 (21%)	5 (18%)
**Positive DSA after tx patients *n* (%)**	36 (52%)	36 (52%)	29 (52%)	29 (62%)	15 (54%)
**ACR after tx patients *n* (%)**					
A2	3 (4%)	3 (4%)	2 (4%)	2 (4%)	0 (0%)
A1	5 (7%)	5 (7%)	5 (9%)	2 (4%)	1 (4%)
A0	61 (88%)	61 (88%)	49 (88%)	43 (91%)	27 (96%)
**CMV mismatched patients *n* (%)**	14 (20%)	14 (20%)	14 (20%)	14 (20%)	14 (20%)
**ICU hours per patient (range)**	147.9 (16–1,434)	147.9 (16–1,434)	162.1 (20–1,434)	148.4 (16–1,434)	184.5 (34–1,434)
**PGD grade patients *n* (%)**					
1	35 (51%)	35 (51%)	27 (48%)	20 (43%)	11 (39%)
2	27 (39%)	27 (39%)	22 (39%)	21 (45%)	11 (39%)
3	5 (7%)	5 (7%)	5 (9%)	5 (11%)	5 (18%)
Unknown	2 (3%)	2 (3%)	2 (4%)	1 (2%)	1 (4%)
**CLAD patients *n* (%)**	13 (19%)	13 (19%)	11 (20%)	13 (28%)	10 (36%)
**Samples *n***	407	365	200	234	185
**Samples per patient *n* (range)**	6 (3–9)	5 (1–9)	4 (1–8)	5 (1–8)	7 (4–9)
**Average sampling days (range)**	207.9 (0–781)	198.4 (0–781)	198.5 (0–755)	210.5 (9–781)	231 (12–781)
**Tx indication patients *n* (%)**					
CF/NCFB	11 (16%)	11 (16%)	8 (14%)	8 (17%)	6 (21%)
CLAD	5 (7%)	5 (7%)	5 (9%)	3 (6%)	3 (11%)
ILD	29 (42%)	29 (42%)	24 (43%)	18 (38%)	10 (36%)
COPD	19 (28%)	19 (28%)	15 (27%)	13 (28%)	7 (25%)
Other^*^	5 (7%)	5 (7%)	4 (7%)	5 (11%)	2 (7%)
**Induction immunosuppression patients *n* (%)**					
Basilimax	34 (49%)	34 (49%)	25 (45%)	24 (51%)	14 (50%)
**Maintenance immunosuppression patients *n* (%)**					
Azathioprine	42 (61%)	42 (61%)	30 (54%)	27 (57%)	16 (57%)
Cyclosporine	1 (1%)	1 (1%)	1 (2%)	1 (2%)	1 (4%)
Everolimus	5 (7%)	5 (7%)	5 (9%)	4 (9%)	5 (18%)
Mycophenolate-Mofetil	44 (64%)	42 (61%)	34 (61%)	27 (57%)	17 (61%)
Prednisolone	69 (100%)	69 (100%)	56 (100%)	47 (100%)	28 (100%)
Tacrolimus	69 (100%)	69 (100%)	54 (96%)	47 (100%)	28 (100%)
**Azithromycin patients *n* (%)**	38 (36%)	36 (35%)	28 (36%)	24 (35%)	18 (39%)
**Antibiotics patients *n* (%)**					
Amoxicillin-clavulanate	5 (7%)	5 (7%)	2 (4%)	4 (9%)	3 (11%)
Trimethoprim-sulfamethoxazole	68 (99%)	68 (99%)	54 (96%)	47 (100%)	28 (100%)
Piperacillin-Tazobactam	14 (20%)	14 (20%)	7 (12%)	4 (9%)	4 (14%)
Tobramycin	11 (16%)	11 (16%)	7 (12%)	5 (11%)	4 (14%)
Other^†^	14 (20%)	13 (19%)	11 (20%)	8 (17%)	9 (32%)
**BAL microbiology patients *n* (%)**					
Negative	68 (62%)	67 (62%)	51 (66%)	46 (64%)	28 (61%)
Positive	41 (38%)	41 (38%)	26 (34%)	26 (36%)	18 (39%)

Data are number (*n*) or percentage (%) of samples or patients and mean with range (min-max). Percentages may not sum to 100% if a patient was administered multiple medications.

ITS, internal transcribed spacer; Tx, transplant; BLT, bilateral lung Tx; SLT, single lung Tx; DSA, donor specific antibodies; DSA at tx is indicative of pre-formed anti-HLA DSA crossed at time of transplant; CMV, cytomegalovirus; ICU, intensive care unit; PGD, primary graft dysfunction. CF, cystic fibrosis; NCFB, non-CF bronchiectasis; CLAD, chronic lung allograft dysfunction; ILD, interstitial lung disease; COPD, chronic obstructive pulmonary disease; BAL, broncho-alveolar lavage.

* Includes patients with combined COPD/pulmonary fibrosis, hypersensitivity pneumoniti,s and sarcoidosis.

† Includes Pentamidine, Meropenem, Ciprofloxacin, Vancomycin.

### 16S rRNA, ITS, RNA sequencing, and metabolomics data analysis

Detailed 16S, ITS, RNA, molecular features, single-cell RNA seq data processing and analysis is outlined in S1 File. 16S and ITS sequencing data was processed using the DADA2 R package. Raw mass-spectrometry (LC-MS) data was processed using the metabolome-lipidome-MS-DIAL pipeline [18]. Molecular features data were annotated using MS-DIAL and the MassBank database [19]. Raw transcriptomics data were analyzed with the nf-core/rnaseq pipeline [20]. Limma was used for differential abundance of bacteria, fungi, and molecular features against time post-transplant in months and transplant indication (false discovery rate, FDR < 0.05, Benjamini–Hochberg (BH) correction), while DESeq2 was used for differential expression of genes (adjusted *p*-value, adj-*p* < 0.05, BH correction). Genes and molecular features trajectories were clustered using the TmixClust package (version 1.24.0). To construct feature trajectories, the log-normalized expression of each gene and molecular feature was scaled and averaged across seven time intervals post-transplant. The TmixClust package was used to cluster feature trajectories along these time intervals. Three distinct trajectories clusters were detected for differentially expressed genes, and 2 were detected for molecular features. The sensitivity analysis was carried out by comparing CLAD patients against all CLAD-free patients available in each dataset, using the same methodology employed for the analysis of the Biomarker cohort. Multi-omics integration was performed using the MOFA+ R package. For the single-cell RNAseq supplementary analysis, publicly available raw FASTQ files from Khatri and colleagues [11] were downloaded from the NCBI Gene Expression Omnibus (GEO) database (accession GSE224210; access token, stwzykkubnkttmd) and reprocessed by the authors using the nf-mucimmuno/scRNAseq pipeline [21].

### Statistical analysis

The lmerTest R package and the splines package were used to test association of Shannon diversity with time post-transplant with a linear mixed-effect natural cubic spline model (df = 3) and patient ID as a random effect. The emmeans package was used to calculate estimated marginal means to derive adjusted effect sizes for time post-transplant and group, while confidence intervals were calculated using the confint function of the stats R package. Associations between transplant indication and Shannon diversity were tested with a Wilcoxon signed-rank test. A *p*-value (p) smaller than 0.05 was considered statistically significant.

## Results

### The microbiome regains diversity and abundance, while *Pseudomonas* associates with previous transplant indication

**Fig 2:**
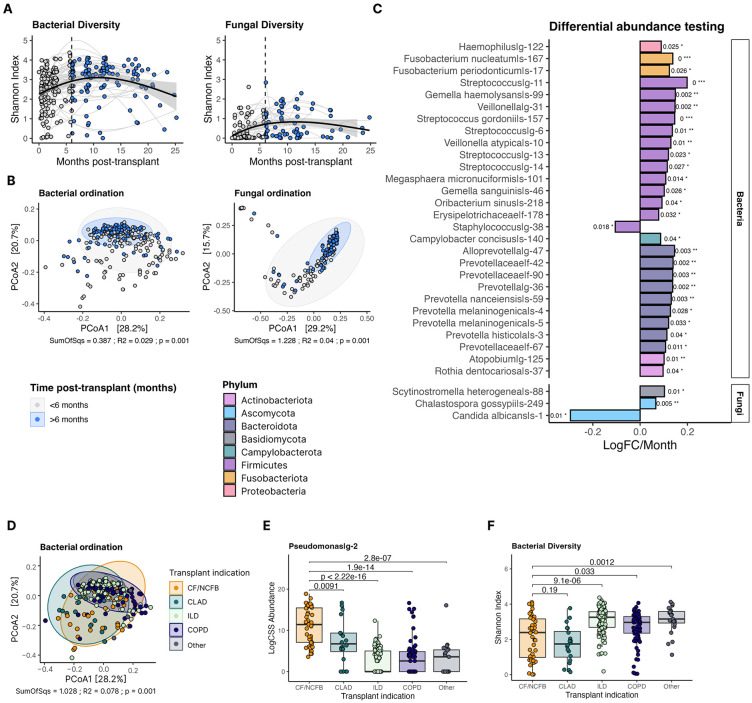
Bacterial and fungal dynamics in CLAD-free patients post-transplant and associations with transplant indication (Stable cohort). **(A)** Scatterplot showing bacterial and fungal predicted Shannon index over months post-transplant (linear model, normal spline with df = 3). Gray shading: 95% confidence interval. Vertical intercept line at 6 months post-transplant. **(B)** Principal coordinate analysis (PCoA) on weighted Unifrac distances showing bacterial and fungal ordination by months post-transplant. Ellipses represent the 95% confidence interval around the group centroid. PERMANOVA test results for time intervals. SumOfSqs (sum of squares): effect size; R2: variance explained, *p*: *p*-value. **(C)** Barplot of Log2 fold changes (Log2FC) for bacterial and fungal amplicon sequence variants (ASVs) showing significant associations with months post-transplant (|Log2FC| > 0, FDR < 0.05). A positive Log2FC reflects an increase over time, a negative value reflects a decrease. **(D)** PCoA on weighted Unifrac distances showing bacterial ordination by transplant indication. PERMANOVA test results for transplant indication. SumOfSqs (sum of squares): effect size; R2: variance explained, *p*: *p*-value. CF/NCFB: cystic fibrosis/non-cystic fibrosis bronchiectasis; CLAD: chronic lung allograft dysfunction; ILD: interstitial lung disease; COPD: chronic obstructive pulmonary disease. **(E)** Boxplots comparing normalized abundance of *Pseudomonas|g-2* across transplant indications (numbers report *p*-values for Wilcoxon signed-rank test). Boxplots are indicative of median, interquartile range (IQR) (boxes), and 1.5× IQR (whiskers). **(F)** Boxplots comparing bacterial Shannon index across transplant indications (numbers report *p*-values for Wilcoxon signed-rank test). Boxplots are indicative of median, interquartile range (IQR) (boxes) and 1.5× IQR (whiskers). Bacteria: 286 samples, 56 CLAD-free recipients; Fungi: 151 samples, 45 CLAD-free recipients.

We first characterized the microbial environment associated with stable CLAD-free allograft function in patient BAL post-transplant (Stable cohort, 0–30 months). The Shannon diversity index increased for both bacteria and fungi during the first 6 months post-transplant (Likelihood ratio test, linear mixed model, bacteria 0.53 increase, 95% CI [0.304,0.757]; *p* < 0.001; fungi 0.45 increase, 95% CI [0.175,0.721]; *p* = 0.0014) (Fig 2A). This temporal transition was also observed for the bacterial and fungal composition. Principal coordinate analysis (PCoA) showed significant separation between time intervals (Fig 2B), indicative of compositional changes post-transplant (PERMANOVA, time interval, bacteria *p* = 0.001, fungi *p* = 0.001). Differential abundance analysis identified 34 bacteria, and 2 fungi associated with time post-transplant (FDR < 0.05) (Fig 2C). Known respiratory genera were significantly increased, such as *Streptococcus*, *Prevotella*, *Alloprevotella*, and *Gemella* [22], while *Staphylococcus* and *Candida albicans* declined. These data suggest that the early post-transplant microbiome is largely dominated by *Staphylococcus* and *Candida Albicans* with diversity and abundance being restored within 6 months.

For the bacterial dataset, we found that previous transplant indication, the total number of antibiotics, time post-transplant, and organ ischemic time could best explain bacterial composition across the entire time post-transplant (S2A Fig). However, total number of antibiotics and organ ischemic time were not linked with any specific differentially abundant bacteria (FDR > 0.05) (S2B and S2C Fig). Transplant indication also significantly impacted the composition of the bacterial microbiome. Samples from patients with cystic fibrosis (CF), non-CF bronchiectasis (CF/NCFB), or undergoing re-transplant (for CLAD) clustered separately from other indications, suggesting a group-specific microbial profile (Fig 2D) (PERMANOVA, transplant indication, *p* = 0.001). They also displayed the highest abundance of *Pseudomonas|g-2* (FDR < 0.05, adj-*p* < 0.001) (Fig 2E) and the lowest Shannon diversity (*p* < 0.05) (Fig 2F). Ischemic time impacted the fungal composition during the first 6 months, while transplant indication had a significant effect only after 6 months (S2D Fig); however, for both clinical variables this was not linked with any differentially abundant fungi (FDR > 0.05). Together, these data highlight relevant environmental and recipient factors associated with altered composition of the microbiota post-transplant.

### Innate immune activity and nitric oxide metabolism reach homeostasis, while T-cell pathways reactivate

**Fig 3:**
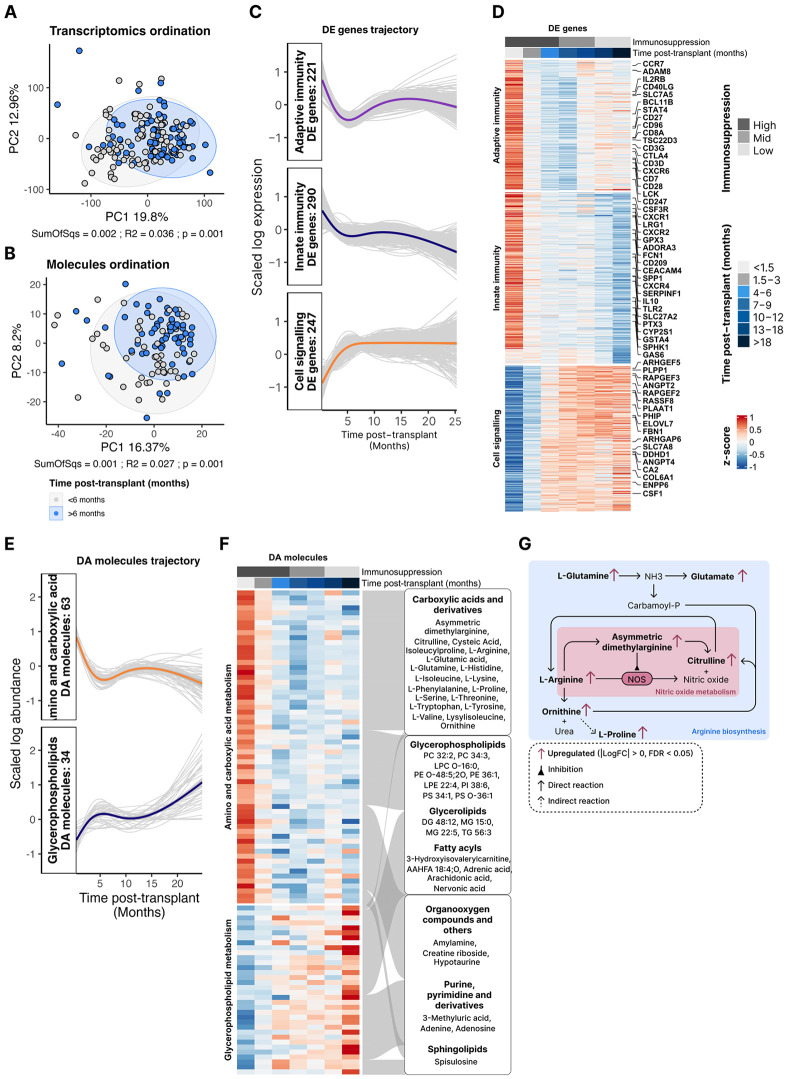
Gene expression, metabolites and lipid dynamics in CLAD-free recipients (Stable cohort). **(A, B)** Principal component analysis (PCA) plot showing gene expression (A) and molecules (B) profile over months post-transplant. Ellipses represent the 95% confidence interval around the group centroid. PERMANOVA test results for time intervals. SumOfSqs (sum of squares): effect size; R2: variance explained, *p*: *p*-value. **(C)** Line plot showing three clusters of average scaled log expression of top differentially expressed (DE) genes (DESeq2, |Log2FC| > 1, adj-*p* < 0.05, 758 DE genes, clustered with TmixClust) over months post-transplant (linear model, normal spline with df = 3). Gray lines show expression trajectory of individual DE genes. **(D)** Heatmap of the average expression of DE genes, with columns arranged by time intervals post-transplant and annotated cluster membership. Due to space limitations, only genes of interest are shown. Z-score indicates scaled log expression for DE genes. **(E)** Line plot showing the average scaled log expression of differentially abundant (DA) metabolite and lipids (molecular features) (Limma, |Log2FC| > 0, FDR < 0.05, 97 DA molecular features, clustered with TmixClust) over time in months post-transplant (linear model, normal spline with df = 3). Gray lines show expression of individual DA molecular features. **(F)** Heatmap of differentially abundant molecular features, with columns arranged by time intervals post-transplant, and annotated cluster and class membership in heatmap rows. Due to space limitations, only molecular features of interest are shown. Z-score indicates scaled log intensity for DA metabolites. **(G)** Schematic showing DA molecular features involved in arginine biosynthesis and nitric oxide metabolism pathway. Differentially abundant molecular features are bolded. Full arrows indicate direct reactions. Dotted arrows indicate indirect reactions. Transcriptomics: 171 samples, 34 CLAD-free recipients; Metabolomics and lipidomics: 116 samples, 18 CLAD-free recipients.

We next analyzed gene expression and small molecules in longitudinal BAL samples from the CLAD-free patients, generated by bulk RNA sequencing and untargeted mass-spectrometry, respectively (Stable cohort, 0−30 months). The small molecule and gene expression datasets showed a significant temporal transition using principal component analysis (PCA) (PERMANOVA, time interval, transcriptomics *p* = 0.001, molecules *p* = 0.001), suggestive of changes in gene expression and molecular profiles in the Stable cohort post-transplant (Fig 3A and 3B). Trajectory clustering separated the top differentially expressed (DE) genes (adj-*p* < 0.05) into 3 groups with diverging expression patterns post-transplant (Fig 3C). The first group of differentially expressed genes (adaptive immunity) showed decreasing expression over the first 3 months, followed by an increase from 12 months onwards, and included genes mapping to several pathways of the adaptive immune system (adj-*p* < 0.05) (S3A Fig). These included T-cell markers *CD3D/E/G*, *BCL11B*, *GZMA/B/K*, *IL2RB*, *CD28*, *CD40LG*, and *LCK*, important for IL-2 expression [23], T cell activation, and cytotoxicity (Fig 3D). The second group (innate immunity) included differentially expressed genes whose expression decreased post-transplant, with pathways belonging to the innate immune system (adj-*p* < 0.05) (S3A Fig). Genes in this group included *CEACAM4*, *CXCR1*, *FCN1*, *TLR2*/*5*, involved in pathogen sensing and phagocytosis. Other genes linked to inflammatory and angiogenic processes of the endothelium, with *GPX3*, responsible for increasing the bioavailability of nitric oxide during oxidative damage [24]; *GAS6*, which enhances the activation of endothelial cells and their interactions with infiltrating immune cells [25]; *LRG1*, *CXCR2/4*, and *SERPINF1* involved in modulating angiogenesis [26–28]; and *CYP2S1*, involved in the conversion of arachidonic acid into pro-inflammatory eicosanoids and prostaglandins [29]. The third group (cell signaling) included differentially expressed genes with increased expression levels over time mapping to general cellular signaling pathways (adj-*p* < 0.05) (S3A Fig), including small GTPases cell signaling genes such as *RAPGEF2/3*, of the Rap subfamily, and *ARHGAP6/24/32* and *ARHGEF5/7* of the Rho subfamily, which are essential regulators of cell survival, proliferation, and adhesion [30]. It also included *CSF1*, linked with macrophage homeostatic injury repair and the resolution of inflammation [31]; *RASSF8* involved in the regulation of tissue repair, proliferation, and migration of epithelial stem cells [32]; and *COL6A6*, indicative of cell-matrix adhesion processes. Others were linked to lipid metabolism, such as the phospholipid sn-1 acyl hydrolase *DDHD1*, the phospholipid phosphatase *PLPP1* and *ENPP6*, a choline-specific phosphodiesterase, all able to interact with different phospholipid classes [33].

Similarly, grouping the differentially abundant molecules’ trajectory (FDR < 0.05) revealed two subsets with opposing trends post-transplant (Fig 3E). In line with the group of innate immunity differentially expressed genes, the amino acids and carboxylic acids subset encompassed molecular features whose abundance declined over time (Fig 3F), mapping to several amino acid metabolism pathways (adj-*p* < 0.05) (S3B Fig). Molecular features included L-arginine, citrulline, asymmetric dimethylarginine, all linked with nitric oxide production [34] (Fig 3G); and arachidonic and adrenic acid, linked with the upregulated expression of pro-inflammatory genes. In line with the group of cell-signaling differentially expressed genes involved in the metabolism of lipids, the second subset (Glycerophospholipids) enriched for glycerophospholipids that increased over time and linked to general lipid metabolic pathways (adj-*p* < 0.05) (S3B Fig). This included unsaturated phosphatidylcholines and phosphatidylethanolamines. Taken together, these results suggest that the first 3 months post-transplant may be a period of declining adaptive and innate immune responses and altered nitric oxide metabolism. These processes are superseded by an increase in homeostatic pathways, and by the reactivation of T-cell genes after 12 months.

### Disrupted glycocalyx and altered sphingolipid metabolism persist prior to the onset of CLAD

**Fig 4:**
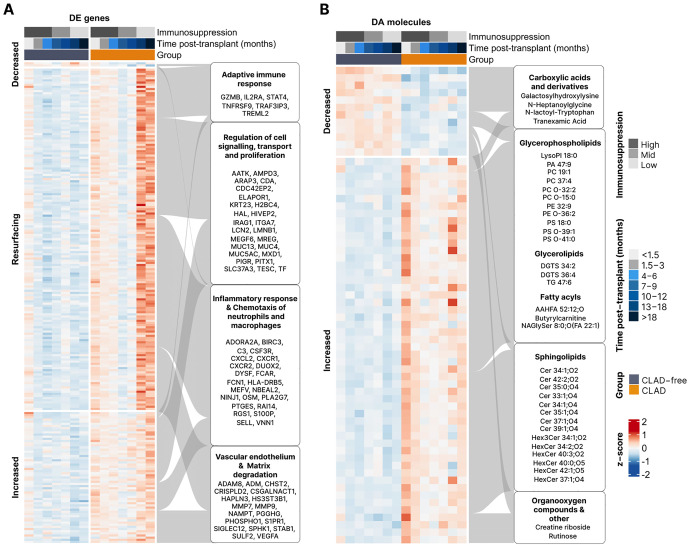
Gene expression, metabolite, and lipids signatures preceding CLAD onset (Biomarker cohort). **(A)** Heatmap of the average expression of differentially expressed genes (Deseq2, |Log2FC| > 1, adj-*p* < 0.05, 222 DE genes, S1 Table), with columns arranged by time intervals post-transplant and annotated whether increased or decreased in CLAD. **(B)** Heatmap of the average expression of differentially abundant metabolites and lipids (Limma, |Log2FC| > 0, FDR < 0.05, 64 DA molecular features, S2 Table), with columns arranged by time intervals post-transplant and annotated whether decreased or increased in CLAD and class membership in heatmap rows. Z-score indicates scaled log expression for DE genes or scaled log intensity for DA metabolites. Due to space limitations, only molecular features of interest are shown. Transcriptomics: 127 samples, 13 CLAD and 13 CLAD-free recipients; Metabolomics and lipidomics: 153 samples, 10 CLAD and 13 CLAD-free patients.

We next sought to compare CLAD-free patients against CLAD patients for the investigation of genes and molecular features associated with the onset of CLAD. We used a matched design strategy to address clinical and demographic heterogeneity in our cohort, matching 13 CLAD patients with 13 CLAD-free patients based on age, sex, and transplant indication (Biomarker cohort 0–30 months post-transplant). Trajectory clustering of differentially expressed genes in the CLAD group (S1 Table) revealed three distinct patterns including genes that were decreased, increased, and those that had a declining trend along the first 6 months post-transplant, followed by a resurfacing after 12 months. Resurfacing differentially expressed genes enriched for pathways linking to the regulation of T cell activation, the regulation of the inflammatory response, and vascular permeability (adj-*p* < 0.05) (S4A Fig). Differentially expressed genes that were increased but showed a more stable pattern linked to pathways of tissue homeostasis and included several genes linked to the vascular endothelium (adj-*p* < 0.05) (S4A Fig). Of note, across both patterns, CLAD patients had significantly increased expression (adj-*p* < 0.05) of genes linked to the glycocalyx, a mechano-sensing proteoglycan layer responsible for protecting the mucin-rich lung epithelium and its vasculature, enabling adhesion of extravasating immune cells [35,36] (Fig 4A). This was exemplified by genes with sulfotransferase activity such as *HAPLN3*, *HS3ST3B1*, *SULF2*, *CHST2/15*, and *CSGALNACT1*, involved in the sulfonation of glycosaminoglycans and chondroitin sulfate, major components of the glycocalyx [35]; as well as matrix remodeling metalloproteinases (*MMP7/9/12*); Other differentially expressed genes support the adhesion and trans endothelial migration of immune cells through the vasculature (*VNN1*, *SELL*) [37], and *IL10*, *CEACAM1/3/5*, *H2AC6*, *CXCR1*/2, *CXCL2* and *CSF3R* with granulocyte chemotactic activity.

Overlaying this transcriptomics signature to a publicly available single-cell RNAseq dataset from CLAD and healthy donor lung tissue [11] showed that a large majority of the differentially expressed genes were expressed by monocytes (S5A and S5B Fig), T and B cells, fibroblasts, and cells of the arterial and lymphatic endothelium (S5C Fig).

The molecules dataset also showed upregulated molecular features (S2 Table) linked with increased vascular permeability, oxidative damage and neutrophil chemotaxis [38], such as Cer 34:1;O2 and several other ceramide species with increasingly elongated fatty-acid chains (Cer 35:1;O4, Cer 37:1;O4, Cer 39:1;O4) (Figs 4B and S4B). Glycerophospholipids were also increased, including 1-alkyl,2-acylglycerophosphocholine and ethanolamine groups (PC O-15:0, PE O-40:5), and diacylglycerophosphocholine, ethanolamine and serine groups (PC19:1, PE 32:9, PS 18:0). Decreased molecular features included carboxylic alpha amino acids such as Galactosylhydroxylysine.

Notably, the CLAD-free group in our Biomarker cohort also demonstrated increased expression of these differentially expressed genes during the first 1.5 months post-transplant (Fig 4A). However, in contrast to the CLAD group, the expression of these genes declined afterwards. Of note, this multi-omics signature was also visible in the Stable cohort during the first 1.5 months (S6A Fig), where it also immediately declined, indicating that it was shared among all CLAD-free patients and was not a unique feature of our CLAD-free Biomarker cohort. Of all differentially expressed genes associated with CLAD, 44 overlapped with the adaptive immunity cluster in the Stable cohort, and 53 with the innate immunity cluster (S6B Fig). In the molecule dataset, 5 metabolites overlapped with the amino acid and carboxylic acid metabolism cluster (S6C Fig), suggestive of shared pathways between the multi-omics signature identified in the Stable cohort and mechanisms associated with CLAD.

We performed an additional sensitivity analysis comparing the results obtained in the Biomarker cohort using 13 matched CLAD-free patients against those obtained by compairing CLAD patients against the full cohort of CLAD-free patients available in each dataset instead (transcriptomics 34 CLAD-free patients, small molecules 18 CLAD-free patients). We found that the majority of the differentially expressed genes and molecules associated with CLAD in our Biomarker cohort maintained their directionality and biological relevance, albeit with reduced effect sizes and significance, due to the introduction of biological heterogeneity and variance from the additional unmatched CLAD-free patients (S1 and S2 Tables).

Both CLAD and CLAD-free groups showed similar increases in Shannon diversity between 1- and 6-months post-transplant (linear mixed model CLAD versus CLAD-free, bacteria, −0.25 increase, 95% CI [−0.906,0.405]; *p* = 0.371; fungi −0.060 increase, 95% CI [−0.519,0.399]; *p* = 0.740) (S7A Fig). There was no evidence that the magnitude of this increase differed between groups (Likelihood ratio test, bacteria *p* = 0.768, fungi *p* = 0.0568), and this was recapitulated for the microbial composition (S7B Fig) and differential abundance testing.

These results suggest that processes preceding CLAD may be tied to the reactivation of the adaptive and innate immune systems, and to the dysregulation of the glycocalyx. This signature appeared visible in all lung transplant recipients during the first 1.5 months post-transplant, suggesting shared processes that resolve in CLAD-free patients but persist in patients that later develop CLAD instead, and intensify as their lung function begins to decline. Crucially, in our cohort, this failure to resolve after 1.5 months is what distinguishes prospective CLAD patients at an early stage post-transplant.

### Data integration reveals key features associated with CLAD onset

**Fig 5:**
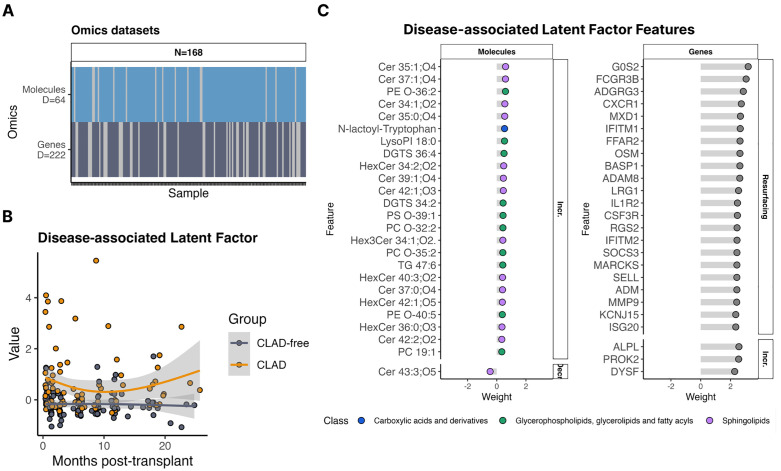
Multi-omics signature integration using MEFISTO (Biomarker cohort). **(A)** Overview of samples (N) in each dataset. Samples are ordered according to time post-transplant in days. “D” indicates the number of DE genes and DA molecules in each dataset. **(B)** Disease-associated Latent Factor values between CLAD and CLAD-free groups over time post-transplant in months. Continuous trend line: predicted values over time in months using a linear model and a normal spline for months (df = 2). Gray shading: 95% confidence interval. **(C)** Top Disease-associated Latent Factor from transcriptomics and proteomics datasets, arranged by decreasing weight value. Positive weight values directly align with disease latent factor pattern and are indicative of high expression in the CLAD group compared to the CLAD-free group. Negative weight values display the opposite pattern (lower in CLAD and higher in CLAD-free).

Next, to understand which features most contributed to the transcriptomics and molecular signatures and shortlist putative candidate biomarkers of CLAD, we submitted all differentially expressed genes and differentially abundant molecules to the MEFISTO framework, including time post-transplant in months as a covariate (Fig 5A, 222 differentially expressed genes and 64 differentially abundant molecules, 13 CLAD and 13 CLAD-free patients). In total, this contained 168 samples across both datasets (127 from the transcriptomics dataset and 153 from the molecules dataset). MEFISTO consolidated all features into a single multi-omics signature (Disease Latent Factor). Within this, the gene expression dataset explained up to 66% of the variation in the data, and the molecules dataset explained 38%. The Disease Latent Factor was significantly associated with CLAD over time post-transplant (linear mixed model, *p* < 0.001). It gradually declined to meet CLAD-free levels only at 12 months and then increased concomitant to the starting decline in lung function and onset of CLAD (Fig 5B). Overview of the top features contributing to the Latent Factor pattern highlighted several sphingolipids, including Cer 34:1;O2 and other ceramide species with longer chains among the top 5, as well as glycerophospholipids (Fig 5C). The top genes contributing to the Latent Factor were differentially expressed genes with a resurfacing trend and included *FCGR3B*, *ADGRG3*, and *CXCR1*, involved in the recruitment and activation of neutrophils, and phagocytosis. Increased differentially expressed genes with a more stable trend post-transplant included *ALPL*, involved in cartilage mineralization, and *PROK2*, involved in endothelial angiogenesis. Taken together, these findings highlight sphingolipids, genes involved in immune cell recruitment, and the endothelial compartment as potential candidate biomarkers associated with the onset of CLAD.

## Discussion

In this prospective study, in the Stable CLAD-free cohort, we identify increased innate and adaptive immune responses and oxidative stress in the first 6 months post-transplant; while in the Biomarker cohort we identify elevated expression of glycocalyx genes and sphingolipids preceding the onset of CLAD.

In the stable CLAD-free cohort, the first 6 months were characterized by the recovery of a diverse respiratory microbiome from one of low diversity dominated by *Staphylococcus* and *Candida.* Since all lung transplant recipients receive broad-spectrum antibiotics prophylaxis during the first 2 weeks post-transplant, we hypothesize these taxa may have exploited their microenvironment as a result of the loss of other taxa induced by the antibiotics, which is in line with previous reports [39–41]. *Pseudomonas* was significantly associated with previous CF/NCFB, complementing reports showing that it repopulates the transplanted allograft in patients with previous cystic fibrosis [22], a process likely to be via re-seeding from microbial reservoirs in the upper respiratory tract. Taken together, our findings suggested that the initial 6 months post-transplant encompassed an unstable state of microbial ecology. We hypothesize that the microbial dominance of *Staphylococcus* and *Candida* is likely to have been responsible for the upregulation of microbial recognition and phagocytosis pathways seen in the transcriptomics dataset.

Additionally, both transcriptomics and molecular feature datasets pointed towards the initial activation of nitric oxide metabolism within the vascular endothelium, suggestive of initial sustained oxidative stress and of compensating homeostatic processes. This was exemplified by the upregulation of genes and molecular features such as Asymmetric dimethylarginine (ADMA), L-Arginine, and L-Citrulline. ADMA inhibits endothelial nitric oxide synthase to halt the production of nitric oxide, and directly competes with L-Arginine, which undergoes oxidation to L-Citrulline and nitric oxide, promoting vascular homeostasis [34]. ADMA has been shown to be predictive of negative outcomes in heart transplants [42], highlighting its potential utility as a marker of oxidative stress. The vascular endothelium is of particular relevance as it is the primary site of alloimmune responses between the recipient’s immune system and the donor tissue during the ischemic injury that occurs upon organ reperfusion [43]. We hypothesize it is largely a residual signature of the ischemia-reperfusion injury process, which appears to resolve in favor of homeostatic processes after 3 months post-transplant. Lastly, the increase in T cell activation genes past 12 months aligned with a gradual lowering of immunosuppression, as per the unit’s protocol, potentially reflecting breakthrough activation of T-cell gene expression.

A dysfunctional endothelial compartment was also a central component of the multi-omics signature that preceded the onset of CLAD in the Biomarker cohort. CLAD patients maintained elevated expression of genes and molecular features linked to the glycocalyx, a hydrophilic protein-rich layer of membrane-binding proteoglycans, glycosaminoglycans, and glycoproteins lining the luminal space of the vascular endothelium [35]. The glycocalyx acts as a shear stress sensor, whereby conformational changes in its components result in increased vascular flow and release of nitric oxide [35], while their degradation and shedding facilitates the adhesion and extravasation of immune cells [44] and the phagocytosis of apoptotic cells [45], resulting in increased alloimmune responses within the graft. In support to our findings, CLAD patients have been found to exhibit a disrupted endothelium upon CLAD onset [46,47]. This was concurrent with elevated BAL hyaluronic acid levels, a glycocalyx component also detected in fibrotic small bronchioles of CLAD explants [48]. The endothelial nature of this signature also explains the epidemiological data that makes ischemia reperfusion injury and primary graft dysfunction a risk factor for CLAD [49].

Mapping the transcriptomics signature to lung tissue revealed most of differentially expressed genes to be expressed by monocytes, T cells, and endothelial cells. Only a subset was expressed by fibroblasts, suggestive of differing mechanisms between CLAD and other tissue remodeling pathologies. Of note, the differentially expressed genes *SPHK1* and *S1PR1* are directly involved in the production of Cer 34:1;O2, which was significantly increased prior to CLAD and, together with other sphingolipid and ceramide species, was also among top features of our integrated multi-omics disease signature. This molecule has been found to be elevated during ischemia reperfusion injury [50], in acute respiratory distress syndrome [51], and in the transplanted lung of single lung recipients post-transplant [13]. More broadly, sphingolipids and ceramides have also been found to increase as a result of neutrophil activity [52] and have been shown to enhance neutrophil chemotaxis and the generation of reactive oxygen species [38]. In the endothelium, *S1PR1 and* ceramides also regulate the egress of T-cells from neighboring lymph nodes [53,54], with the potential for exacerbated alloimmune responses to the graft.

Given the sustained activation and the resurfacing dynamics of this signature, we propose it to be a biomarker characteristic of patients that achieve a CLAD diagnosis within the first 3 years post-transplant, in which patients are primed to develop CLAD during the first 6 months post-transplant. Crucially, our results also suggest the best time to identify such incipient CLAD patients to be after 1.5 months post-transplant, since both groups appear to share this signature during this time frame.

We acknowledge that an important limitation of this hypothesis-generating study is the lack of an independent validation cohort, which restricts the generalizability of our results. In addition, our patient matching approach reduces the effective sample size and may have resulted in limited power to detect more modest effects, as seen in our microbiome analysis, which did not return significant differences between our groups. Given the observational nature of our study, our findings are therefore to be considered associative and not aimed at establishing causality. Moreover, given the immune nature of the transcriptomics signature, we hypothesize that its dynamics could have been modulated by the concurrent corticosteroid and immunosuppression regime. Another limitation of this study is that we could not access information on the exact dosage prescribed to our patients, hence the relationship between dosage and signature expression could not be established. We furthermore did not account for cell compositional heterogeneity in our analyses. Nevertheless, these results provide invaluable insights into the processes associated with the onset of CLAD and suggest genes and molecular features to be further validated in upcoming cohorts.

Future studies incorporating targeted approaches (such as quantitative polymerase chain reaction (PCR) and targeted mass-spectrometry), as well as multi-omics profiling of independent patient cohorts—particularly also including patients diagnosed beyond 3 years post-transplant—are needed to confirm our observations, assess reproducibility across sub-populations, and strengthen the clinical relevance of the proposed candidate biomarkers. Following validation, key predictive features could be used to assess patients post-transplant, enabling the adjustment of current therapies and inform the development of novel approaches against CLAD.

In conclusion, we have identified endothelial and immunological processes preceding CLAD development, and key metabolites, lipids, and genes associated with its onset. These results enhance our understanding of homeostatic adaptation processes that prevail in patients with stable lung function, and the pathological mechanisms that may underlie the development of CLAD, which could critically influence the success and survival of lung allografts.

## Supporting information

S1 Fig Sample collection and clinical testing. **(A)** Schematic of longitudinal sample collection. **(B–D)** Schematic of recorded instances of azithromycin usage, donor-specific antibodies (DSA) testing, acute cellular rejection result, and cytomegalovirus (CMV) in BAL and blood. The y-axis represents an individual patient. Samples from the same patient are connected along the x-axis and CLAD diagnosis is marked.
(TIFF)

S2 Fig Bacterial and fungal associations with clinical metadata (Stable cohort). **(A–D)** Bar plots of bacterial (A) and fungal (D) PERMANOVA results tested. R2% explained corresponds to the percentage of variation explained by the factor of the model. The number on each bar plot corresponds to the p-value for a specific factor (* adj-*p* < 0.05, ** adj-*p* < 0.01). **(B, C)** Principal coordinate analysis (PCoA) on Unifrac distances showing bacterial ordination by total number of antibiotics (B) and ischemic time in minutes (C). Ellipses represent the 95% confidence interval around the group centroid. PERMANOVA test results for number of antibiotics (B) and ischemic time (C). SumOfSqs (sum of squares): effect size; R2: variance explained, *p*: *p*-value.
(TIFF)

S3 Fig Pathway and metabolism analysis (Stable cohort). **(A)** Pathway analysis result showing significant pathways in each cluster (clusterProfiler, adj-pval < 0.05), in decreasing order of significance. **(B)** Pathway analysis result showing significant pathway per cluster (FELLA, adj-pval < 0.05), in decreasing order of significance.
(TIFF)

S4 Fig Pathways and metabolism analysis (Biomarker cohort). **(A)** Pathway analysis result showing significant pathways in each cluster (clusterProfiler, adj-pval < 0.05), in decreasing order of significance. **(B)** Schematic showing CLAD-associated DA metabolites, lipids, and DE genes involved in the glycerophospholipid and ether lipid metabolism, and the sphingolipid metabolism. Differentially abundant molecules are bolded. Full arrows indicate direct reactions. Dotted arrows indicate indirect reactions.
(TIFF)

S5 Fig Transcriptomics signature associated with CLAD (Biomarker cohort). **(A)** Donor and CLAD tissue UMAP generated by authors using publicly available single-cell RNA sequencing raw data from Khatri and colleagues, *JCI Insight* 2023, showing annotated immune and epithelial cell clusters. Raw data is available on GEO under accession GSE224210; access token stwzykkubnkttmd. **(B)** Transcriptomics CLAD-associated signature scoring showing DE genes expression by cell type (Deseq2, |Log2FC| > 1, adj-*p* < 0.05, 222 DE genes), with a higher score corresponding to higher expression by that cell type. **(C)** Heatmap of transcriptomics CLAD-associated signature showing DE gene expression by cell type and class. Due to space limitations, only genes of interest are shown.
(TIFF)

S6 Fig Differentially expressed genes and differentially abundant molecules overlap between Stable and Biomarker study cohorts. **(A)** Heatmap of differentially abundant genes and molecules, with columns arranged by study cohorts, time interval post-transplant, and groups. Rows indicate whether DE gene or DA molecule overlapped with adaptive, innate immune and cell signaling cluster membership from the transcriptomics and metabolomics analysis of the stable cohort (see Fig 3). Z-score indicates scaled log expression for DE genes or scaled log intensity for DA metabolites. **(B)** Venn diagrams showing DE genes (Deseq2, |Log2FC| > 1, adj-*p* < 0.05, 222 DE genes) overlap between Stable and Biomarker study cohorts. **(C)** Venn diagrams showing DA molecules (Limma, |Log2FC| > 0, FDR < 0.05, 64 DA molecules) overlap between Stable and Biomarker study cohorts.
(TIFF)

S7 Fig Bacterial and fungal associations with CLAD (Biomarker cohort). **(A)** Line plots showing bacterial and fungal predicted Shannon index between groups over months post-transplant (linear model, normal spline with df = 3). Gray shading: 95% confidence interval. **(B)** Weighted Unifrac distance PCoA plot showing group overlap post-transplant for bacteria and fungi. Ellipses represent the 95% confidence interval around the group centroid. PERMANOVA test results for group. SumOfSqs (sum of squares): effect size; R2: variance explained, *p*: *p*-value. Bacteria 156 samples, 13 CLAD and 13 CLAD-free; fungi 107 samples, 11 CLAD and 13 CLAD-free.
(TIFF)

S1 File Supplemental file containing supplementary methods and materials used for sample processing and data analysis. (DOCX)

S1 Table Supplementary table reporting all differentially abundant genes associated with CLAD and sensitivity analysis results. (CSV)

S2 Table Supplementary table reporting all differentially abundant molecules associated with CLAD and sensitivity analysis results. (CSV)

S3 Table Supplementary table reporting MS-DIAL height intensity output for all features detected in the metabolomics dataset (positive mode). (CSV)

S4 Table Supplementary table reporting MS-DIAL height intensity output for all features detected in the metabolomics dataset (negative mode). (CSV)

S5 Table Supplementary table reporting MS-DIAL height intensity output for all features detected in the lipidomics dataset (positive mode). (CSV)

S6 Table Supplementary table reporting MS-DIAL height intensity output for all features detected in the lipidomics dataset (negative mode). (CSV)

S1 Protocol Study protocol including ethics committee certificate of approval, study protocol, and analysis plan with version changes. Project number 430/17.
(PDF)

S1 STROBE Checklist Completed STROBE checklist statement for cohort studies. The STROBE checklist is best used in conjunction with this article (freely available on the Web sites of PLoS Medicine at http://www.plosmedicine.org/, Annals of Internal Medicine at http://www.annals.org/, and Epidemiology at http://www.epidem.com/). Information on the STROBE Initiative is available at http://www.strobe-statement.org.
(DOCX)

## Abbreviations

ADMA: asymmetric dimethylarginine
BALs: broncho-alveolar lavages
BH: Benjamini–Hochberg
BOS: bronchiolitis obliterans
CF: cystic fibrosis
CLAD: chronic lung allograft dysfunction
DA: differentially abundant
DE: differentially expressed
DSA: donor-specific antibodies
FEV1: forced expiratory volume in 1 s
PCA: principal component analysis
PCR: polymerase chain reaction
PCoA: principal coordinate analysis
PGD: primary graft dysfunction
RAS: restrictive allograft syndrome
STROBE: Strengthening the Reporting of Observational Studies in Epidemiology
