# Supplementary figures and images for: Multi-omics biomarkers of endothelial dysregulation preceding chronic lung allograft dysfunction: A prospective cohort study
Source: PLoS Med. 2026 Jun 23;23(6):e1004725. doi: 10.1371/journal.pmed.1004725 (PMC13289948; doi:10.1371/journal.pmed.1004725)

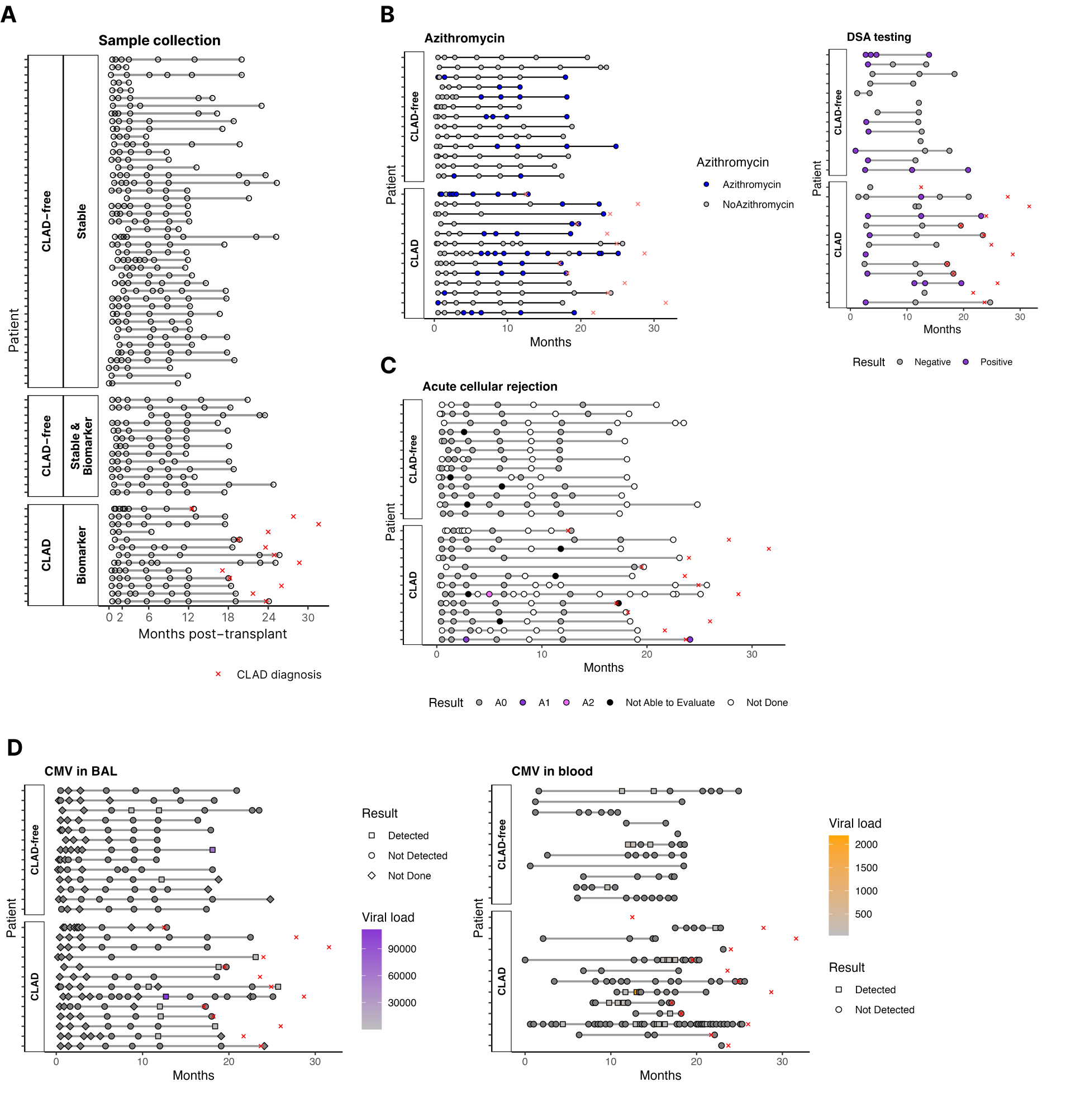

Supplement: S1 Fig — (A) Schematic of longitudinal sample collection. (B–D) Schematic of recorded instances of azithromycin usage, donor-specific antibodies (DSA) testing, acute cellular rejection result, and cytomegalovirus (CMV) in BAL and blood. The y-axis represents an individual patient. Samples from the same patient are connected along the x-axis and CLAD diagnosis is marked. (TIFF) [file pmed.1004725.s001.tiff]

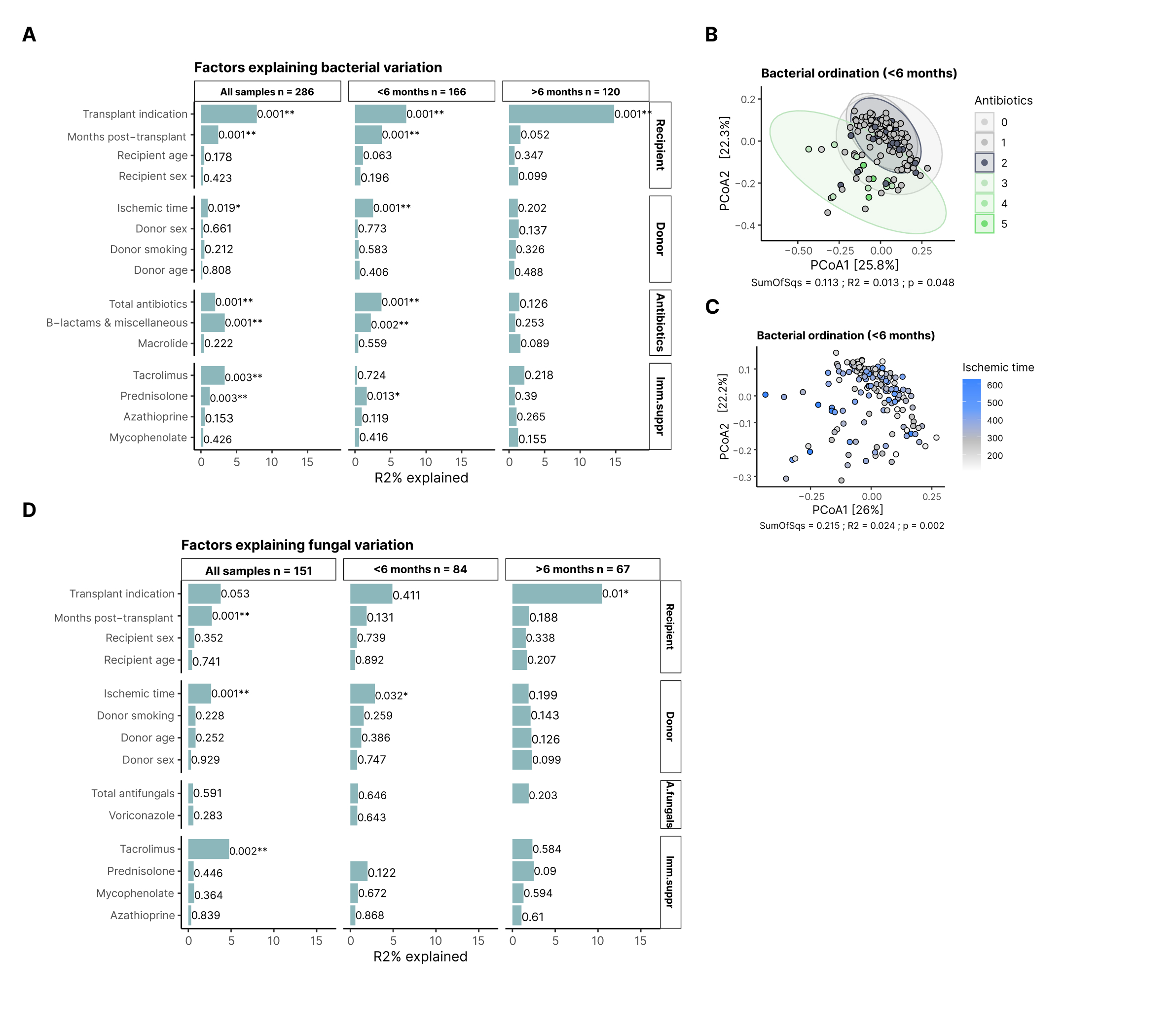

Supplement: S2 Fig — (A–D) Bar plots of bacterial (A) and fungal (D) PERMANOVA results tested. R2% explained corresponds to the percentage of variation explained by the factor of the model. The number on each bar plot corresponds to the p-value for a specific factor (* adj-p < 0.05, ** adj-p < 0.01). (B, C) Principal coordinate analysis (PCoA) on Unifrac distances showing bacterial ordination by total number of antibiotics (B) and ischemic time in minutes (C). Ellipses represent the 95% confidence interval around the group centroid. PERMANOVA test results for number of antibiotics (B) and ischemic time (C). SumOfSqs (sum of squares): effect size; R2: variance explained, p: p-value. (TIFF) [file pmed.1004725.s002.tiff]

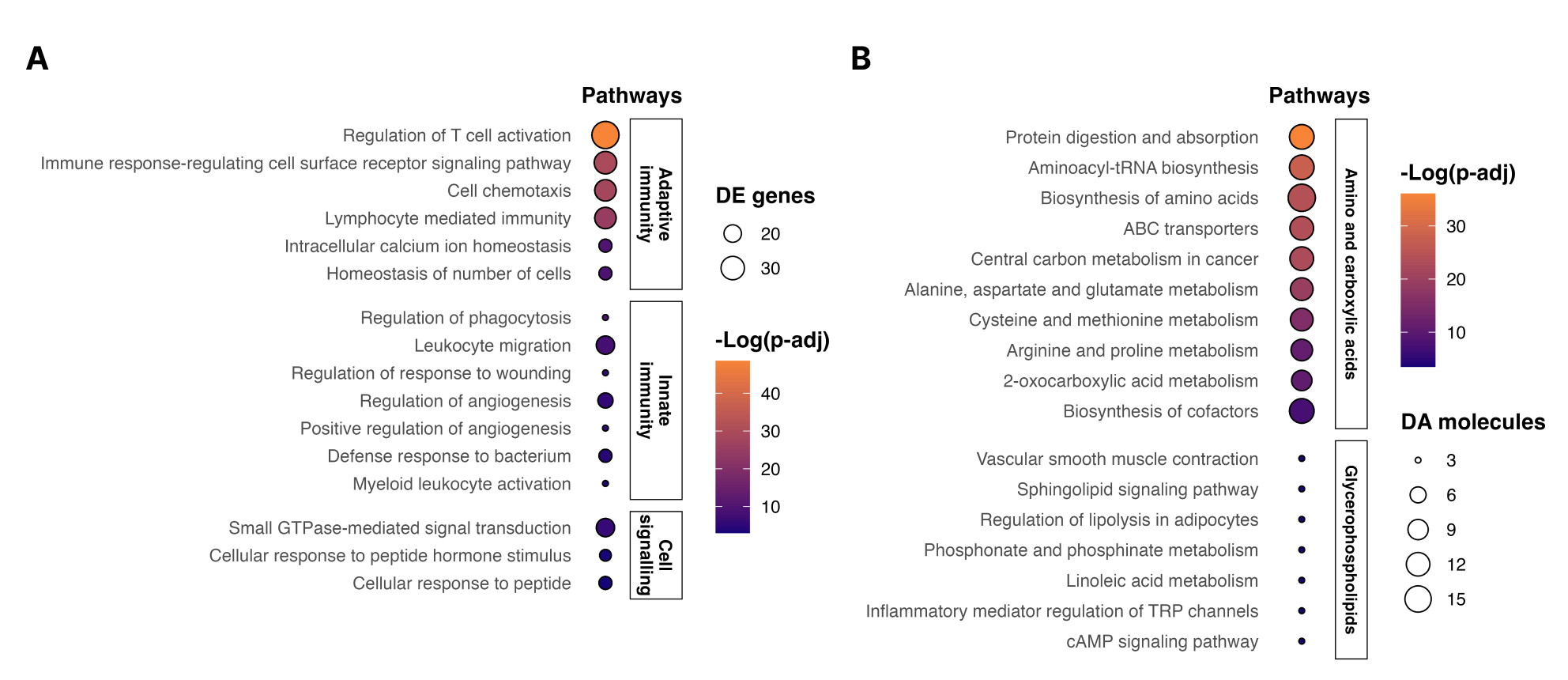

Supplement: S3 Fig — (A) Pathway analysis result showing significant pathways in each cluster (clusterProfiler, adj-pval < 0.05), in decreasing order of significance. (B) Pathway analysis result showing significant pathway per cluster (FELLA, adj-pval < 0.05), in decreasing order of significance. (TIFF) [file pmed.1004725.s003.tiff]

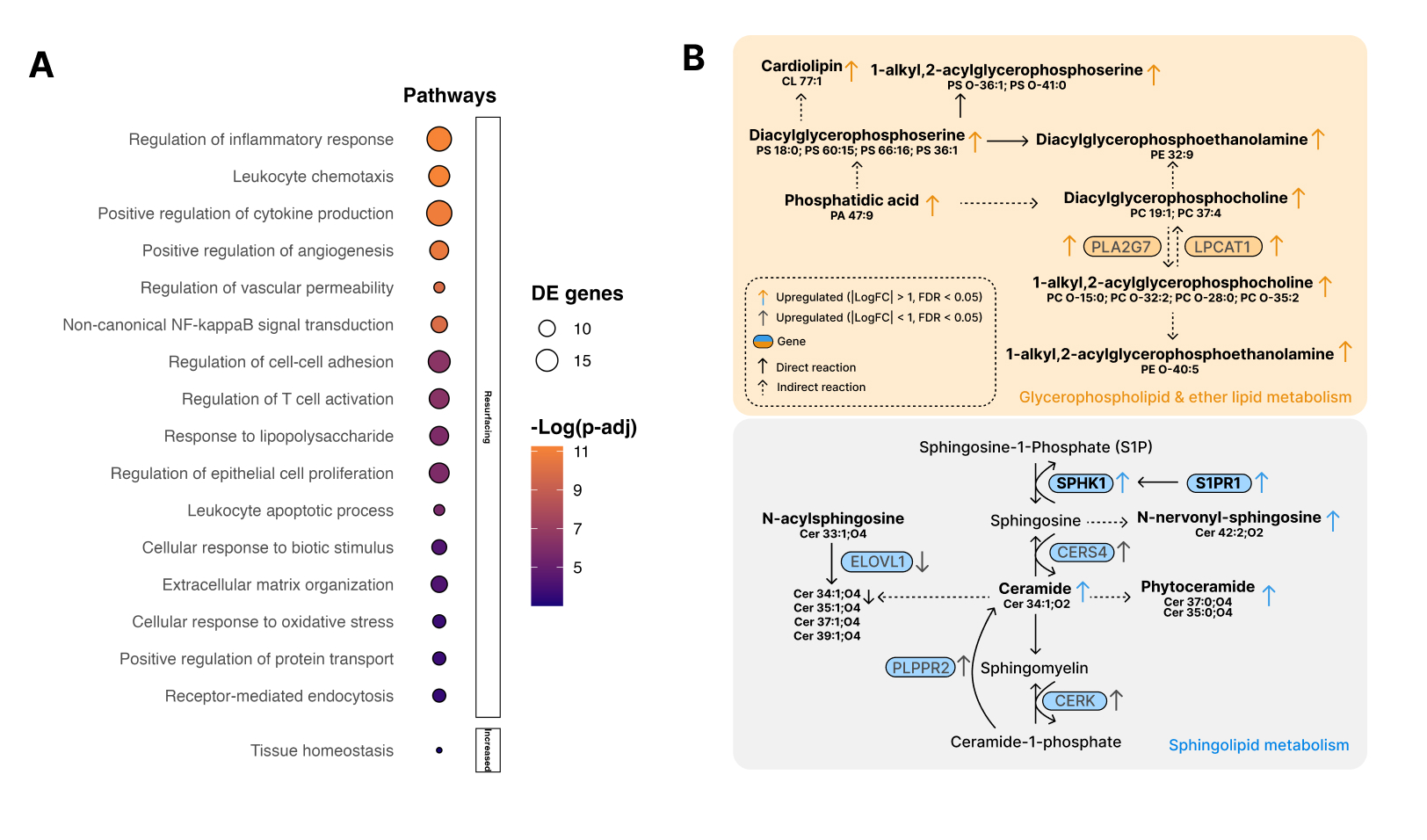

Supplement: S4 Fig — (A) Pathway analysis result showing significant pathways in each cluster (clusterProfiler, adj-pval < 0.05), in decreasing order of significance. (B) Schematic showing CLAD-associated DA metabolites, lipids, and DE genes involved in the glycerophospholipid and ether lipid metabolism, and the sphingolipid metabolism. Differentially abundant molecules are bolded. Full arrows indicate direct reactions. Dotted arrows indicate indirect reactions. (TIFF) [file pmed.1004725.s004.tiff]

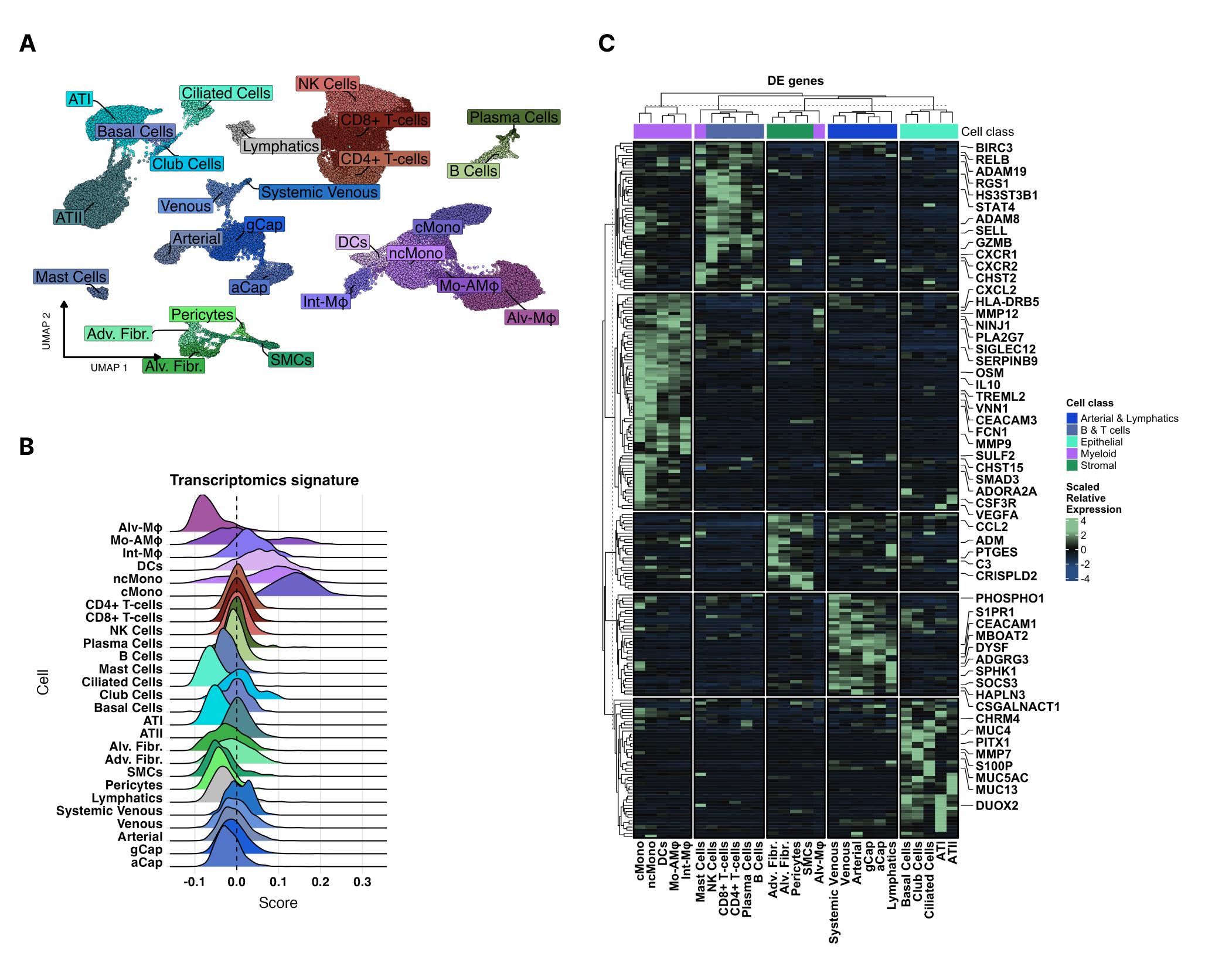

Supplement: S5 Fig — (A) Donor and CLAD tissue UMAP generated by authors using publicly available single-cell RNA sequencing raw data from Khatri and colleagues, JCI Insight 2023, showing annotated immune and epithelial cell clusters. Raw data is available on GEO under accession GSE224210; access token stwzykkubnkttmd. (B) Transcriptomics CLAD-associated signature scoring showing DE genes expression by cell type (Deseq2, |Log2FC| > 1, adj-p < 0.05, 222 DE genes), with a higher score corresponding to higher expression by that cell type. (C) Heatmap of transcriptomics CLAD-associated signature showing DE gene expression by cell type and class. Due to space limitations, only genes of interest are shown. (TIFF) [file pmed.1004725.s005.tiff]

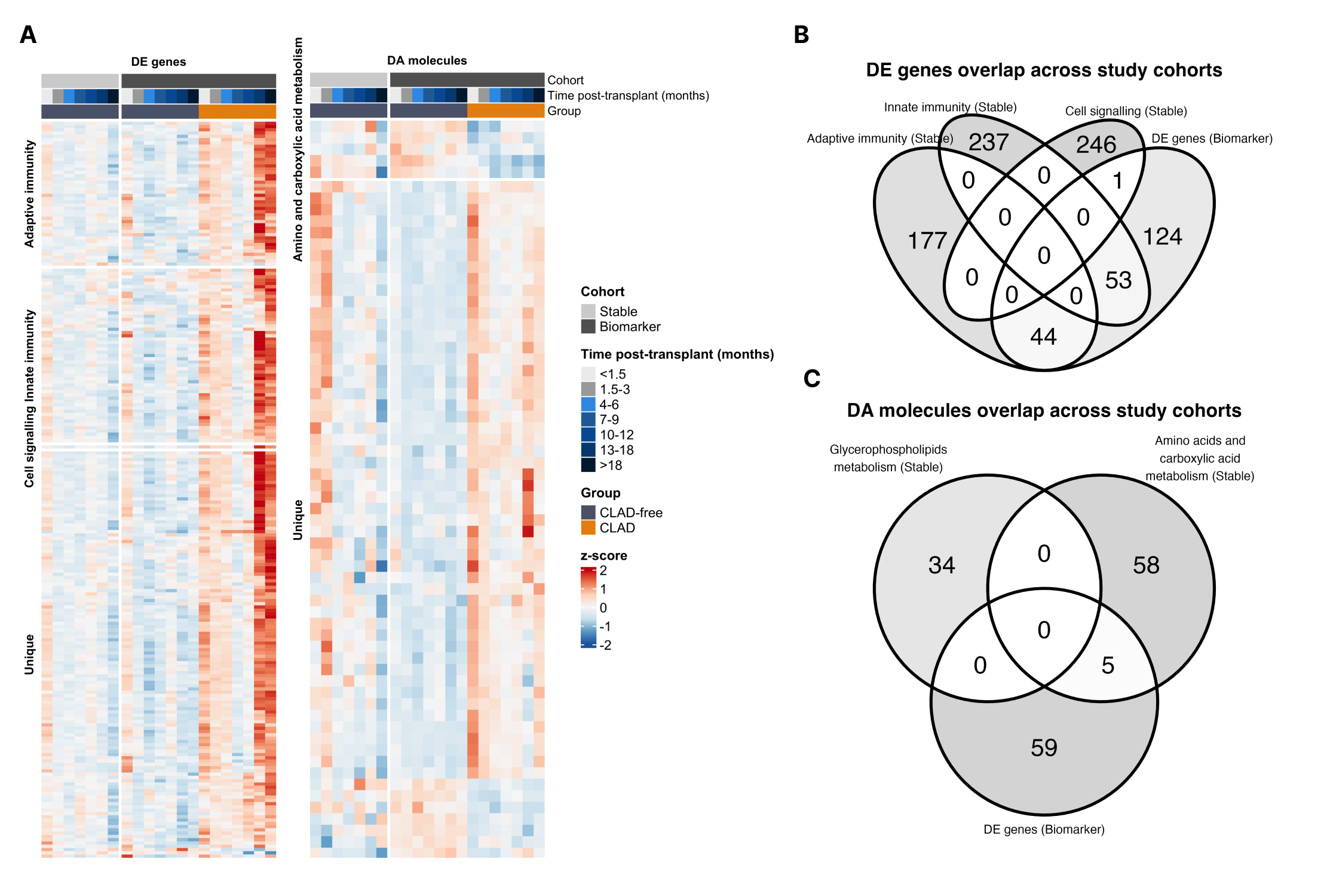

Supplement: S6 Fig — (A) Heatmap of differentially abundant genes and molecules, with columns arranged by study cohorts, time interval post-transplant, and groups. Rows indicate whether DE gene or DA molecule overlapped with adaptive, innate immune and cell signaling cluster membership from the transcriptomics and metabolomics analysis of the stable cohort (see Fig 3). Z-score indicates scaled log expression for DE genes or scaled log intensity for DA metabolites. (B) Venn diagrams showing DE genes (Deseq2, |Log2FC| > 1, adj-p < 0.05, 222 DE genes) overlap between Stable and Biomarker study cohorts. (C) Venn diagrams showing DA molecules (Limma, |Log2FC| > 0, FDR < 0.05, 64 DA molecules) overlap between Stable and Biomarker study cohorts. (TIFF) [file pmed.1004725.s006.tiff]

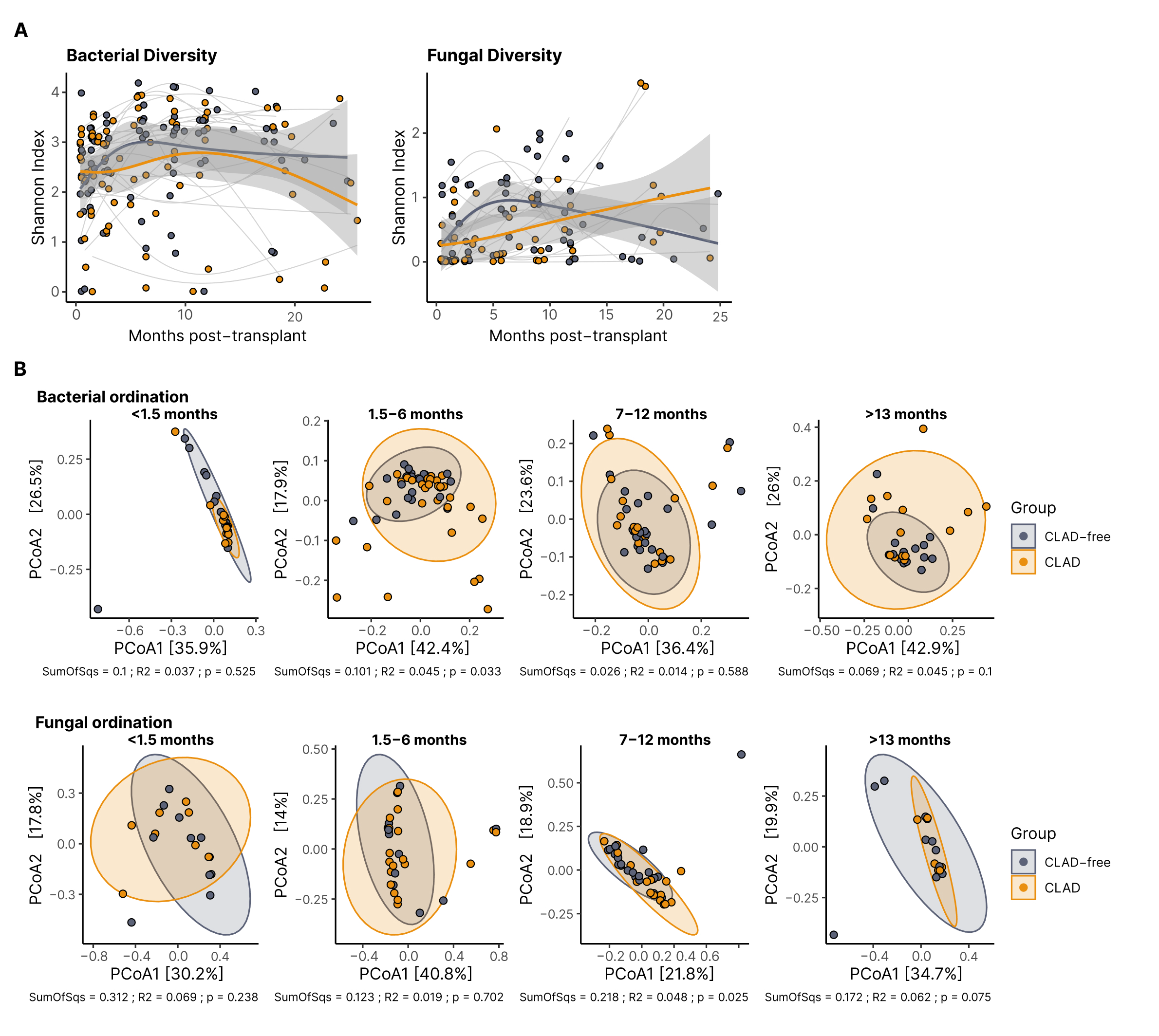

Supplement: S7 Fig — (A) Line plots showing bacterial and fungal predicted Shannon index between groups over months post-transplant (linear model, normal spline with df = 3). Gray shading: 95% confidence interval. (B) Weighted Unifrac distance PCoA plot showing group overlap post-transplant for bacteria and fungi. Ellipses represent the 95% confidence interval around the group centroid. PERMANOVA test results for group. SumOfSqs (sum of squares): effect size; R2: variance explained, p: p-value. Bacteria 156 samples, 13 CLAD and 13 CLAD-free; fungi 107 samples, 11 CLAD and 13 CLAD-free. (TIFF) [file pmed.1004725.s007.tiff]
