# Supplementary material for: Multi-omics biomarkers of endothelial dysregulation preceding chronic lung allograft dysfunction: A prospective cohort study
Source: PLoS Med. 2026 Jun 23;23(6):e1004725. doi: 10.1371/journal.pmed.1004725 (PMC13289948; doi:10.1371/journal.pmed.1004725)
Supplement: S1 File — (DOCX) [file pmed.1004725.s008.docx]

**Supplementary material**

**Multi-omics biomarkers of endothelial dysregulation preceding chronic lung allograft dysfunction: A prospective cohort study**

Giulia Iacono^1^, Christina Begka^1^, Bailey Cardwell^1^, Carmel Daunt^1^, Roxanne Chatzis^1^, Celine Pattaroni^1^, Alana Butler^1^, Matthew Macowan^1^, Bronwyn Levvey^2^, Gregory I. Snell^2^, Glen P. Westall^1,2&^, Benjamin J. Marsland^1&^

^1^Department of Immunology, School of Translational Medicine, Monash University, Melbourne, Australia

^2^Lung Transplant Service, Department of Respiratory Medicine, Alfred Hospital, Melbourne, Victoria, Australia

^&^Co-Senior Authors.

**1. Statistical analysis plan**

***Description of patient cohort***

Between March 2018 and March 2022, a total of 69 lung transplant recipients were included into this study at the Alfred Hospital (Melbourne, Victoria Australia). All clinical metadata for this study was collected prospectively in a REDCap database and included information on recipients’ age, gender, transplant indication, microbiology, antimicrobial and antiviral therapy, and immunosuppressive therapy. This study was approved by the Alfred Hospital Ethics Committee (ID 430/17). Formal written informed consent was provided by all patients.

***Patient recruitment***

- Inclusion criteria:
  - Recipient of bilateral or single lung transplantation between March 2018 and March 2022
  - Absence of CLAD at 30 months post-transplant (Stable cohort)
  - Diagnosed with CLAD by 30 months post-transplant (Biomarker cohort)
- Exclusion criteria:
  - Less than 2 samples passed quality control steps per single patient
  - Insufficient FEV1 assessments recorded post-transplant
  - Withdrawn from study
  - Diagnosed with CLAD (Stable cohort)
  - Did not provide informed consent form
  - FEV1 decline but patient failed to achieve CLAD diagnosis before 30 months post-transplant (Biomarker cohort)

No power calculation was performed for this explorative study. The study sample size is indicative of all eligible patients that fulfilled the inclusion criteria during the study period.

***Hypotheses***

Stable cohort analysis: A concurrent longitudinal analysis of the transplanted respiratory microbiome, transcriptome and metabolome will reveal shared homeostatic processes relevant for stable long-term lung function post-transplant.

Biomarker cohort analysis: The onset of CLAD is associated with a multi-omics signature detectable before a significant decline in lung function.

***Study objectives***

Stable cohort analysis: To describe bacterial, fungal, metabolomic, lipidomic, and gene expression trajectories post-transplant in patients who remained free of chronic lung allograft dysfunction (CLAD).

Biomarker cohort analysis: To identify bacteria, fungi, metabolites, lipids and genes whose increase or decrease precedes the onset of CLAD and identify key predictive features of CLAD.

Response variables: Shannon diversity, beta diversity, normalized bacterial abundance, normalized molecules intensities, normalized gene expression.

Explanatory variables: time post-transplant, transplant indication, disease group.

***Pharmacological management post-transplant***

Pharmacological management post-transplant was conducted as per the Alfred Hospital transplant post-operative guidelines. All patients received standard immunosuppression post-transplant, including a calcineurin or mTOR inhibitor (Tacrolimus, Cyclosporine or Everolimus), an antiproliferative medication (Azathioprine or Mycophenolate Mofetil), and a steroid (Prednisolone). Tacrolimus trough levels were maintained at 10–12 ng/mL during the first 0-6 months post-transplant, reduced to 8–10 ng/mL between 6–12 months, and 5–8 ng/mL beyond 12 months. Cyclosporine trough levels were maintained at 250–300 ng/mL within the first 0–6 months, 200–250 ng/mL between 6–12 months, and 100–200 ng/mL after 12 months. Prednisolone doses were tapered from 0.25 mg/kg within the first 3 months to 0.2 mg/kg between 3–6 months, 0.15 mg/kg between 6–12 months, and 7.5 mg daily after 12 months. Mycophenolate mofetil was administered at 2000 mg daily (15 mg/kg if <50 kg), Azathioprine at 1.5 mg/kg daily, and Everolimus trough levels were maintained between 4–8 ng/mL. Trough levels represent the concentration of a medication detected prior to the administration of the next dose. Patients at risk of renal dysfunction received induction therapy with Basiliximab. At-risk patients received Valganciclovir for at least 11 months post-transplant. Patients were administered intravenous antibiotics for the first 7 to 14 days post-transplant, typically using the broad-spectrum b-lactam antibiotics (Piperacillin-tazobactam), unless donor/recipient microbiology results suggested the need for an alternative antimicrobial therapy. Azithromycin treatment was started in patients who experienced early microbial infections beyond the first 7-days post-lung transplant. Patients did not receive any antifungal prophylaxis. At two weeks post-transplant, patients started co-trimoxazole (Trimethoprim-sulfamethoxazole) prophylaxis against *Pneumocystis*, and Valganciclovir prophylaxis against *Cytomegalovirus*. Patients with de novo DSA were managed according to the degree of accompanying lung allograft dysfunction. Treatment approaches included: no change to baseline immunosuppression (n = 34); changing Azathioprine to Mycophenolate mofetil (n = 6); starting monthly IVIg (0.4g/kg) (n =10); Methyl prednisolone (n = 4); Plasmapheresis and Rituximab (n = 1). The study was performed at a time that the unit’s immunosuppressive regimen was transitioning from Azathioprine to Mycophenolate mofetil. On the advice of the nephrologists, one patient with BK viremia was converted from tacrolimus to azathioprine. mTOR inhibitors were introduced in patients with renal dysfunction to either reduce (n = 2) or replace tacrolimus (n = 4).

***Bronchoscopies, lung function tests and CLAD assessment***

Surveillance bronchoscopy was performed at 2, 6 weeks, 3, 6, 9, 12, 18 months post-transplant. Clinically indicated bronchoscopies were performed after 18 months or whenever advised by the clinicians until study termination at 30 months post-transplant. Sample collection was omitted in the case where a patient was assessed as temporarily unfit to undergo the bronchoscopy procedure. In addition, sample collection was delayed or restricted during the height of the COVID pandemic (March 2020 - July 2021). Linear modelling along time post-transplant in months was used to handle missing sample data. Pulmonary function tests were performed regularly coinciding with each patient’s lung transplant clinic review. Biopsies collected for acute cellular rejection were scored by a pathologist at the Alfred Hospital.

**2. Methods and materials**

***BAL collection and processing***

Bronchoalveolar lavage (BAL) was performed under sterile conditions. For BAL collection, a flexible bronchoscope was inserted through the nasal cavity and 2x 50ml normal sterile saline (0.9%) was delivered to either right middle or left lingular lobe and subsequently aspirated and recovered. To account for microbial environmental traces originating from the procedure room, 5 negative controls involving a sterile bronchoscope saline flush were also collected throughout the duration of this study and processed with the same methods as BAL samples. Post-aspiration, BAL was kept at 4°C and processed within 2h of collection. Under a sterile laminar flow hood, 1ml was centrifuged at 2,000xg for 10min at 4°C. The supernatant was separated into another tube and used for metabolomics and lipidomics analysis. The pellet of two tubes was combined and resuspended in 350μl RLT buffer (79216, Qiagen) and vortexed for 10 seconds. All supernatants and pellets were snap frozen on dry ice and stored at -80°C. For microbiome analysis, 1.5ml was centrifuged at 14,000xg for 10min at 4°C. Cell pellets were snap frozen on dry ice and stored at -80°C.

***BAL preparation***

*16S and ITS DNA extraction and amplicon library preparation*

DNA extraction was performed under a PCR hood using sterile and microbial DNA/DNAse-free material. DNA was isolated using the DNeasy UltraClean Microbial Kit (12224-50, Qiagen) according to the manufacturer’s protocol, with the addition of a lyticase (SRE0018-200KU, Sigma-Aldrich) incubation step for fungal DNA extraction. DNA was eluted in 40μl microbial DNA-free water (338132, Qiagen). Microbial environmental contamination was controlled using negative extraction controls using microbial DNA-free water. Extraction efficiency was controlled using ZymoBIOMICS Microbial Community Standard (D6300, Integrated Sciences) positive controls.

Extracted microbial DNA was amplified using custom barcoded PCR primers linked to an Illumina adaptor, targeting the V1-V2 hypervariable region of the bacterial 16S rRNA gene (F-27/R-338), and the internal transcribed spacer (ITS) region of the fungal ITS gene. Primers were as following, with N sequences as sample-specific 12-nucleotides golean barcodes:

16S-Forward:

5’-AATGATACGGCGACCACCGAGATCTACACTATGGTAATTCCAGMGTTYGATYMTGGCTCAG-3’;

16S-Reverse:

5’-CAAGCAGAAGACGGCATACGAGATACGAGACTGATTNNNNNNNNNNNNAAGCTGCCTCCCGTAGGAGT-3’;

ITS-Forward:

5’-AATGATACGGCGACCACCGAGATCTACACGGCTTGGTCATTTAGAGGAAGTAA-3’;

ITS-Reverse:

5’-CAAGCAGAAGACGGCATACGAGATNNNNNNNNNNNNCGGCTGCGTTCTTCATCGATGC-3’

Each 25μl PCR reaction was composed of 5μl of DNA (or negative or positive extraction control), 15.4μl of microbial DNA-free water, 1μl of forward and reverse primers at 5μM, 2.5μl of Accuprime PCR buffer II, 0.1μl Accuprime TAQ High Fidelity DNA polymerase (12346086, Life Technologies). Each run included 2 negative reaction controls using microbial DNA-free water (338132, Qiagen). PCR was performed under a PCR hood using sterile and microbial DNA/DNAse-free material. Cycling parameters: initial denaturation 3 min at 94°C, followed by 40 cycles of 30 s denaturation at 94°C, 30 s annealing at 56°C and 60 s elongation at 68°C, with a final extension at 68°C for 10min. Amplified PCR products were quantified by capillary electrophoresis using a High Sensitivity next generation sequencing (NGS) Fragment Analysis Kit (ATI-DNF-474-0500, Integrated Sciences) and a 12-capillary Fragment Analyser System (5200, Agilent). PCR amplicons were pooled at equimolar concentrations of 4nM and purified using Agencourt AMPure XP beads (A63881, Beckman Coulter). Denatured amplicon libraries were sequenced using the MiSeq Illumina technology (MiSeq Reagent Kit v2 - 500-cycles, 2x 250bp, 20% PhiX) at Monash STM Genomics in Melbourne.

*Transcriptomics*

For BAL transcriptomics, RNA was extracted using the Quick-RNA MiniPrep Kit (R1055, Integrated Sciences), according to the manufacturer’s protocol. RNA was quantified by capillary electrophoresis using a High Sensitivity NGS Fragment Analysis Kit (ATI-DNF-472-0500, Integrated Sciences) and a 12-capillary Fragment Analyser System (5200, Agilent). Samples were sequenced on an Illumina Novaseq6000 at Novogene, Singapore. The NEBNext Ultra RNA Library Prep Kit for Illumina NovaSeq PE150 and ribosomal RNA depletion was used for sequencing batch 1, while the NEBNext Ultra Directional RNA Library Prep Kit for Illumina with Poly-A tail enrichment was used for transcriptomics batches 2 and 3. Batch effects due to different library preparation methods were accounted for during batch correction steps prior to the analysis (see transcriptomics raw data processing).

*Metabolomics and lipidomics*

Samples were thawed and centrifuged at 800g for 5min at 4C to remove any residual cell debris. 100μl of supernatant was used for concurrent metabolomics and lipidomics extraction. Blanks were made with water (338132, Qiagen) and extracted alongside. 400μl cold extraction solvent (containing 0.5uM CCTP internal std, 5uM BHT, 1/1000 15N, 13C-Amino Acid mixture in 3:1, v/v Methanol (1060351000, Sigma-Aldrich)/Chloroform (650498, Sigma-Aldrich) was added to the supernatant, and samples were mixed at 4C for 1h. Samples were then centrifuged at 20,000g for 10min. The supernatant was evaporated for 30min until dry under a nitrogen stream at 20C. Samples were re-solubilized in 100μl CHCl_3_ : MeOH : H_2_O (1 : 3 : 1, v/v) for metabolomics and BuOH : MeOH : H_2_O (4.5 : 4.5 : 1, v/v spiked with lipid internal standards) for lipidomics. Samples were sonicated with ice for 15 min, centrifuged at 14.8g for 10min at 4C and transferred to mass spectrometry vials. Supernatant leftovers were combined to make a pooled QC sample. Samples were randomized to account for signal drift and acquired on a QExactive mass spectrometer (Thermo Scientific) at the Monash Proteomics and Metabolomics Facility in Parkville, Melbourne Victoria Australia. Metabolomics and lipidomics samples were acquired in two separate mass-spectrometry runs specifically optimized for the acquisition of the different types of molecules.

**3. Data analysis**

*Microbiome raw data processing*

Raw sequencing data was processed using the dada2 pipeline using the DADA2 (version 1.14.1) R package. Source code for raw microbiota data processing can be obtained at: <https://github.com/respiratory-immunology-lab/microbiome-dada2>. Briefly, samples were demultiplexed using the iu-demultiplex (version 2.7) function from illumina-utils tools, cutadapt (version 2.10) was then used to remove primers and adaptors in ITS samples. Forward and reverse reads were truncated using *truncLen = c(240, 240)* for bacteria and *c(0,0)* for fungi, *maxEE = c(2,2) and trunQ = c(2,2).* The remaining parameters were set as default. Bacterial taxonomy was assigned against the SILVA 16S rRNA database (version 139). Fungal taxonomy was assigned against the ITS UNITE general release database (10.05.2021 version). Sequences were aligned using the *DECIPHER* R package (version 2.18.1). The *phangorn* R package (version 2.5.5) was then used to construct a maximum likelihood phylogenetic tree using the neighbour-joining tree as a starting point. In order to minimize the impact of microbial contamination, the R package *Decontam* (version 1.12.0) was used to identify and remove contaminant amplicon sequence variants (ASVs) using the prevalence method (16S > 0.5, ITS > 0.3). To minimize the effect of trace amounts of bacteria and fungi, any samples and controls below a minimum read filter of 200 reads were first removed. Any ASVs that could not be classified to a Phylum were also removed before applying *Decontam*. 53 bacterial and 27 fungal ASVs were classified as contaminants and removed from the dataset. To account for variability between sequencing runs, the *tip_glom* function from the *phyloseq* package was used to agglomerate taxa based on phylogenetic tree distance (h = 0.02, hcfun = “agnes”). Taxa below a count threshold of 1 were subsequently filtered out using the *phyloseq* package. Finally, a total read threshold of 2000 reads per sample was then applied and samples below the threshold were excluded. For both bacterial and fungal datasets, none of the negative controls (PCR, DNA extraction and bronchoscope flush) passed the filter, except for one DNA extraction control in the fungal dataset. The 16S dataset included 1992 ASVs and 365 samples. The ITS dataset included 603 ASVs and 200 samples. Bacterial ASVs were distributed among 18 phyla with Firmicutes, Bacteroidota, Actinobacteriota and Proteobacteria as the most abundant; and 221 genera, including *Streptococcus, Prevotella, Actinomyces, Leptotrichia, Fusobacterium* as the most abundant. Fungal ASVs were distributed among 2 phyla, Basidiomycota and Ascomycota; and 196 genera, including *Candida, Aspergillus, Phlebia, Vishniacozyma, Athelia,* as the most abundant.

*Microbiome data analysis*

The Shannon alpha-diversity index was calculated using the *phyloseq* R package (version 1.46.0). Association of Shannon diversity with time post-transplant was tested using a linear mixed-effect natural cubic spline model (df = 3) with the *lmerTest* package (version 3.1-3) and the splines R package (version 4.4.2) with patient ID as a random effect. Estimated marginal means were calculated using the *emmeans* R package (version 1.11.1). Estimated marginal means were used to derive adjusted effect sizes, calculated as differences in predicted outcomes between groups or time points. The overall effect of time was evaluated using a likelihood ratio test comparing the full model including the spline term to a reduced model without the time term. Confidence intervals were calculated using the *confint* function from the stats R package (version 4.3.2). For beta-diversity analysis and differential abundance (DA) analysis, microbial datasets were filtered using the core function from the *microbiome* R package (version = 1.24.0, abundance = 15, prevalence = 0.3 for bacteria and 0.01 for fungi). Datasets were then cumulative sum scaled normalized, and log transformed (LogCSS) using the *metagenomeSeq* R package (version 1.43.0). The *Limma* R package (version 3.58.1) was used to detect differentially abundant ASVs over months post-transplant using a normal spline (df = 3) in the stable cohort, and between groups over months post-transplant (normal spline, df = 3) in the biomarker cohort, while accounting for patient ID as random effect using the *duplicateCorrelation* function. In each cohort, this linear mixed model was adjusted for transplant indication, antibiotics usage and organ ischemic time. Investigation of factors explaining microbial variation was performed using the permutational analysis of variance (PERMANOVA) method using the *adonis2* function of the *vegan* package (version 2.6-4) on weighted Unifrac dissimilarities (999 permutations). Given the heterogeneity of the antibiotics prescribed, we grouped medications based on their mode of action. This resulted in 4 subgroups, including B-lactams (such as Amoxicillin-clavulanate, Piperacillin-Tazobactam), macrolide (Azithromycin), sulfonamide-benzylpyrimidine (such as Trimethoprim-sulfamethoxazole), and miscellaneous ones in a miscellaneous category.

*Transcriptomics raw data processing*

Raw FASTQ files were processed using the nf-core/rnaseq pipeline (version 3.14.0) [20]. Quality control was performed using *FastQC*, and reads trimming was performed using Trim Galore. Genomic contaminants and ribosomal components were removed with *BBSplit* and *SortMeRNA* respectively. STAR and Salmon tools were used to align and quantify reads, which were sorted and indexed using *SAMtools*. A re-estimated counts matrix was generated abundance matrix and used for downstream analysis. The *filterByExpr* function from the *edgeR* package (version 4.2.1) was used to remove lowly expressed genes. *CombatSeq* from the *sva* R package (version 3.50.0) was used to correct the data for sequencing batches. Y-chromosome related genes, including the *XIST* gene, and non-protein coding genes were removed. After filtering, the transcriptomics dataset contained 12707 genes.

*Transcriptomics data analysis*

Principal component analysis (PCA) was computed using the *prcomp* function of the *stats* R package (version 4.3.2). *DESeq2* was used to detect differentially expressed (DE) genes (adjusted p-value (adj-p) < 0.05) over months post-transplant using a normal spline (df = 3) in the stable cohort and between groups over months post-transplant (normal spline, df = 3) in the biomarker cohort. In each cohort, this linear model was adjusted for transplant indication, antibiotics usage and organ ischemic time. The Log2 fold change of DE genes identified was shrunk using the *ashr* shrinkage method (*ashr* R package, version 2.2-63). Prior to visualization, counts were normalized using the *vst* function from the *DESeq2* R package (version 1.42.0). Clustering of DE genes was performed using the *TMixClust* R package (version 1.24.0). To infer gene trajectories over time, the log normalized expression of each gene was scaled and averaged across seven time intervals post-transplant. Gene ontology pathway analysis was performed using the *clusterProfiler* R package (version 4.14.6).

*Metabolomics and lipidomics raw data processing*

LC-MS data was processed using the metabolome-lipidome-MSDIAL pipeline [18]. Raw Thermo Fisher sample files were deconvoluted, aligned and annotated using MS-DIAL (version 4.7). Data was annotated against the MassBank negative and positive databases (version 2021.04). Parameters used were *MS tolerance* = 0.005, *min peak height* = 100’000, *Rt time tolerance* = 2min, *gap filling* = TRUE. The raw data matrix of the alignment output was used for downstream analysis. The *pmp* R package (version 1.4.0) was used to filter samples by blanks (*fold_change = 5, fraction_in_blank = 0.3*). Observations were filtered for prevalence across samples using the filter_peaks_by_fractions function (*min_frac = 0.3, method = ‘across’*), and within (quality control) QC samples with the filter_peaks_by_rsd (*max_rsd = 65*). The filtered dataset was probabilistic quotient normalized, missing values were imputed using the “random forest” method of imputation, and data was scaled using the generalized logarithm method. The Combat function of the *sva* R package (version 3.40.0) was used to correct for batch effects. Metabolites and lipids were annotated using the Lipid MAPS, GNPS, HMDB and MS-DIAL databases. All successfully annotated features were manually screened for raw spectra quality within MS-DIAL, resulting in 448 metabolites and 537 reliable lipids for downstream analysis.

*Metabolomics and lipidomics data analysis*

PCA was computed using the *prcomp* function of the *stats* R package (version 4.3.2). The *Limma* R package was used to detect differentially abundant metabolites and lipids over months post-transplant using a normal spline (df = 3) in the stable cohort and between groups over months post-transplant (normal spline, df = 3) in the biomarker cohort, while accounting for patient ID as random effect using the *duplicateCorrelation* function. In each cohort, this linear mixed model was adjusted for transplant indication, antibiotics usage and organ ischemic time. To infer molecules trajectory over time, the log normalized expression of each molecule was scaled and averaged across seven time intervals post-transplant. Clustering was performed using the *TMixClust* R package (version 1.24.0). The *FELLA* R package (version 1.26.0) was used to perform pathway analysis using available KEGG IDs.

*Data visualization*

Best-fit curves were fitted by predicting microbial abundance, gene expression and metabolite intensity over months post-transplant using *geom_smooth* of the ggplot package (version 3.5.1)*.* Smoothed mean and confidence intervals (95%) were estimated by the geom_smooth function when plotting. Significance for fixed effects was calculated using the *lmerTest* package (version 3.1-3). Heatmaps were plotted using the *ComplexHeatmap* package (version 2.18.0). Overlap between genes and metabolites was plotted using the *ggVennDiagram* package (version 1.5.2).

*Single cell RNA sequencing analysis*

Public raw FASTQ files from Khatri et. al 2023 [11] were downloaded from the NCBI Gene Expression Omnibus (GEO) database (accession GSE224210; access token, stwzykkubnkttmd). Using the nf-mucimmuno/scRNAseq pipeline [21], they were trimmed to remove low quality reads with *trimgalore* (version 0.6.10) specifying a minimum quality of 20 and read length of 26. Read quality was assessed before and after using *FastQC* (version 0.12.1). Trimmed reads were aligned to the human genome (GRCh38 version 111) using *STARsolo* (version 2.7.10b) with parameters recommended by the *STARsolo* developers for alignment of 10X 3' V3 data akin to CellRanger output. Gene-level summarized count tables from the "GeneFull_Ex50pAS" output from *STARsolo* were imported into R for further analysis. Background contamination was removed with *SoupX* (version 1.6.2). Low quality cells (<1000 features, <3000 UMI counts, >25% mitochondrial reads, <0.8 log10(genes/UMI)) were removed from further analysis. Putative doublets were identified with *DoubletFinder* (version 2.0.4) and removed. The remaining cells were normalized with Seurat's *SCTransform* (version 5.0.1) on a per-batch (patient) basis using 5000 highly variable features. Patient-specific batch-effects were then removed using CCA of the Seurat R package and a UMAP was constructed from the corrected CCA dimensions. Cells passing all QC thresholds were mapped to the human lung cell atlas (HLCA), which acted as a baseline reference to infer cluster identity. Louvain clusters and HLCA annotations were compared side by side along with a set of genes aggregated from our own internal scRNAseq datasets, canonical markers of cell types, and several previously published ones (all of the latter are included as part of the HLCA). Spurious clusters of cells with contradictory cell markers without specificity to a disease state were manually removed from further analysis. From this, Louvain clusters were manually assigned ensuring consistent labelling from the HLCA and the manual determination. The transcriptomics signature was scored onto the CCA-batch corrected dataset following correction for differences in library sizes between batches with the PrepSCTFindMarkers() function. The average expression per annotated cluster was extracted and used as input to create a heatmap of expression values for genes of interest. The signature score of genes of interest was calculated using the *AddModuleScore* function in Seurat.

*MEFISTO analysis*

Multi-omics integration with molecules and gene expression datasets was performed using the MEFISTO option from the MOFA+ R package (version 1.16), including months as a time covariate (convergence_mode = slow, num_factors = 1). Missing sample profiles were imputed using the impute() function from the same package. Association of Disease Factor with Group and time post-transplant was tested using a linear mixed-effect natural cubic spline model (df = 3) with the lmerTest package (version 3.1-3) and the splines R package (version 4.4.2) with patient ID as a random effect.

All analyses were performed using R (version 4.4.2).
