# Supplementary material for: Multi-omics biomarkers of endothelial dysregulation preceding chronic lung allograft dysfunction: A prospective cohort study
Source: PLoS Med. 2026 Jun 23;23(6):e1004725. doi: 10.1371/journal.pmed.1004725 (PMC13289948; doi:10.1371/journal.pmed.1004725)
Supplement: S1 Protocol — Project number 430/17. (PDF) [file pmed.1004725.s015.pdf]

[Alfred logo]

ETHICS COMMITTEE CERTIFICATE OF APPROVAL

This is to certify that

Project No: 430/17

Project Title: Identifying Immune and Infectious Biomarkers of Allograft Dysfunction Following Lung Transplantation

Principal Researcher: A/Professor Glen Westall

Protocol Version 1.1 **dated:** 21-Aug-2017

Participant Information and Consent Form Version 1 **dated:** 12-Sep-2017

*was considered by the Ethics Committee on **21-Sep-2017**, meets the requirements of the National Statement on Ethical Conduct in Human Research (2007) and was **APPROVED** on **22-Sep-2017***

It is the Principal Researcher's responsibility to ensure that all researchers associated with this project are aware of the conditions of approval and which documents have been approved.

The Principal Researcher is required to notify the Secretary of the Ethics Committee, via amendment or progress report, of

- Any significant change to the project and the reason for that change, including an indication of ethical implications (if any);
- Serious adverse effects on participants and the action taken to address those effects;
- Any other unforeseen events or unexpected developments that merit notification;
- The inability of the Principal Researcher to continue in that role, or any other change in research personnel involved in the project;
- Any expiry of the insurance coverage provided with respect to sponsored clinical trials and proof of re-insurance;
- A delay of more than 12 months in the commencement of the project; and,
- Termination or closure of the project.

Additionally, the Principal Researcher is required to submit

- A Progress Report on the anniversary of approval and on completion of the project *(forms to be provided)*;

The Ethics Committee may conduct an audit at any time.

All research subject to the Alfred Hospital Ethics Committee review must be conducted in accordance with the National Statement on Ethical Conduct in Human Research (2007).

The Alfred Hospital Ethics Committee is a properly constituted Human Research Ethics Committee in accordance with the National Statement on Ethical Conduct in Human Research (2007).

SPECIAL CONDITIONS

None

SIGNED:

Professor John J. McNeil
Chair, Ethics Committee

Please quote project number and title in all correspondence

Specific Aims #3, #4 and #5 indicated in study protocol relate to the aims and data collected for this manuscript.

Alfred Ethics Project Number: 430 / 17

IDENTIFYING IMMUNE AND INFECTIOUS BIOMARKERS OF ALLOGRAFT DYSFUNCTION FOLLOWING LUNG TRANSPLANTATION

Protocol Date: **21st August 2017**
Version Number: **1.1**

Principal Investigator: A/Prof Glen Westall

Co-Investigators: Prof Greg Snell (Alfred Health), Dr Miranda Paraskeva (Alfred Health), Dr Helen Whitford (Alfred Health), Dr Kovi Levin (Alfred Health), Dr Sakhee Kotecha (Alfred Health), Prof Trevor Williams (Alfred Health), A/Prof Bronwyn Levvey (Alfred Health), Prof David Tarlinton (Monash University), Prof Ben Marsland (Monash University), Prof Anton Peleg (Alfred Health), Dr Orla Morrissey (Alfred Health), Dr Lucy Sullivan (Alfred Health and University of Melbourne)

CONTENTS:

1. Abbreviations 2
2. Summary 3
3. Hypothesis 4
4. Broad Project Aims 4
5. Background 4
6. Study Proposal 6
7. Research Plan 7
8. Methodology 9
9. Inclusion/Exclusion Criteria 11
10. Analysis 11
12. References 12

1. ABBREVIATIONS

ADCC	Antibody-Dependent Cell-Mediated Cytotoxicity
AFOP	Acute Fibrinoid Organizing Pneumonia
AMR	Antibody Mediated Rejection
BAL	Broncholaveolar Lavage
BOS	Bronchiolitis Obliterans Syndrome
CLAD	Chronic Lung Allograft Dysfunction
DAD	Diffuse Alveolar Damage
DSA	Donor Specific Antibodies
HLA	Human Leukocyte Antigens
LTx	Lung Transplant
MFI	Mean Fluorescence Intensity
MHC	Major Histocompatability Complex
NK	Natural Killer
OB	Obliterative Bronchiolitis
PCRs	Polymerase Chain Reactions
RAS	Restrictive Allograft Syndrome
Tfh	Follicular Helper T Cells

2. SUMMARY

Transplantation saves the lives of patients with end stage lung disease. Better operative techniques, anaesthesia, organ preservation and more potent immunosuppression have improved outcomes significantly but long-term graft survival remains unchanged. Transplanted lungs are lost due to an ill-defined process called chronic lung allograft dysfunction (CLAD) that is characterized by either fibrosis of the airways (bronchiolitis obliterans syndrome = BOS) or of the lung architecture (restrictive allograft syndrome = RAS) (1). CLAD is characterised by a drop in lung function, that once established is irreversible. The major risk factors for CLAD are i) acute rejection as diagnosed on lung biopsy and ii) infection, particularly CMV and aspergillus. To date, biomarkers have not been identified that can diagnose acute rejection or predict the early onset of CLAD.

We have previously explored a number of biomarkers and demonstrated an association with allograft dysfunction following lung transplant (LTx). We have shown that activin and follistatin levels vary with time from LTx and reflect a proinflammatory environment (2,3). We continue to explore the role of NK cells following LTx having previously shown that NK cells become activated in patients undergoing acute rejection or CMV infection (4, 5). Collaborative research with the Doherty Institute has demonstrated a link between immune cells that control CMV and the later development of CLAD (6). We now wish to extend this biomarker approach with a new prospective cohort of LTx patients who will provide blood and BAL samples to allow further interrogation of immune and infectious links to allograft dysfunction following LTx.

Identifying, CLAD at an early stage, may allow therapeutic interventions to interrupt the progression to irreversible damage to the transplanted lung. We propose to analyse in both a cross-sectional and longitudinal study whether a series of biomarkers can predict for episodes of acute rejection or the development of CLAD in a cohort of patients undergoing LTx at the Alfred Hospital.

3. HYPOTHESIS

The identification of biomarkers of immune function and infection in the blood and lung will predict episodes of acute rejection and the onset of chronic lung allograft dysfunction (CLAD) following LTx.

4. BROAD PROJECT AIMS

4.1. To establish the immune response in the serum and broncholaveolar lavage (BAL) as measured cross-sectionally and longitudinally during the first year post-LTx and the association with later episodes of acute rejection or the subsequent development of CLAD.

4.22. To determine whether episodes of viral, fungal or bacterial infection in the LTx recipient trigger further increases in the secretion of these biomarkers and thus act as an inflammatory stimulus.

5. BACKGROUND

5.1 The immune response to lung transplantation

Successful outcomes following LTx depend on our ability to pharmacologically manipulate the immune system to prevent rejection of the lung allograft. The antigens involved in graft rejection are encoded by the major histocompatability complex (MHC). This group of highly polymorphic genes defines the human leukocyte antigens (HLA) which are expressed on virtually all cell surfaces. The polymorphic nature of these allopeptides defines the genetic signature of each individual and allows the host immune system to differentiate between self and foreign peptides. The majority of transplanted lung allografts are MHC-mismatched to the recipient. As such, the transplanted lung will be seen as foreign to the recipient's immune system. This leads to activation of immune pathways that if left unchecked would lead to rejection and destruction of the newly transplanted lung. We are interested in whether structural differences between transplant recipients and their donors, termed eplets, may drive the acute cellular and humoral rejection responses following LTx.

5.2 Chronic Lung Allograft Dysfunction

LTx chronic rejection has been described pathologically as Obliterative Bronchiolitis (OB) and physiologically as airflow limitation, in the form of the Bronchiolitis Obliterans Syndrome (BOS). However, increasingly other phenotypes of chronic rejection have been recognized, in particular the Restrictive Allograft Syndrome (RAS), characterized by restrictive physiology, interstitial infiltrates on radiology, and interstitial fibrosis, acute fibrinoid organizing pneumonia (AFOP) and diffuse alveolar damage (DAD) on histology (REF). To encompass these important emerging variants and concepts of chronic dysfunction, the term Chronic Lung Allograft Dysfunction (CLAD) has recently been created. We have been interested to see whether structural differences between transplant recipients and their donors may drive

immune responses that lead to the later development of CLAD. Historically, the degree of matching between a transplant recipient and donor has been determined by counting the number of mismatched HLA-A, B and DR antigens of the donor. HLA antigens have multiple epitopes that can be recognised by specific antibodies. Elucidation of the three-dimensional molecular structures and amino acid sequence differences between HLA antigens allows structural characterisation of HLA antigens, and what are termed eplets. We have recently demonstrated that HLA matching at the eplet level protects against CLAD (7).

5.3 Acute Rejection

Despite the use of maintenance immunosuppression, acute cellular rejection is a common problem post lung transplant with 34% of adult LTx recipients experiencing at least one episode during the first year post-transplant. The lung allograft is particularly susceptible to alloreactivity with the incidence of acute rejection being higher following LTx compared to other solid organ transplants. Whilst the direct effects of acute rejection are usually controlled and reversed following high-dose intravenous steroid treatment, the indirect effects remain problematic, with episodes of acute rejection being strongly associated with the later development of chronic lung allograft dysfunction (CLAD). Given the need for invasive transbronchial biopsies to provide a histological diagnosis of acute cellular rejection, we have previously evaluated biomarkers in the blood and BAL and their association with acute rejection (2-5). Functional assays of T cell and B cell function in the blood may identify patients at higher risk of acute cellular rejection. Analysis of the BAL is very useful in providing mechanistic insights into the immunology of graft function and rejection following LTx. However, BAL findings seen in acute rejection must be interpreted in the context of those described in stable lung transplant recipients, and after taking into account the confounding nature of chronic airway infection.

5.5 Infection in immunosuppressed lung transplant recipients

Infection is commonly encountered following LTx and is largely related to the use of immunosuppressive medication. We have a long-standing interest in how opportunistic infections such as CMV (8) and Aspergillus (9) have both direct effects on the lung allograft, as well as indirect effects through an association with CLAD. The airways, historically thought to be sterile, are now known to harbor a microbiota that changes in its constituents depending upon the health status of an individual. This lung microbiome consists of bacteria, viruses and

fungi, which are in intimate contact with cells in the lung providing tonic stimuli to our immune system. As is routine clinical practice, the current study will also include a detailed prospective analysis of infections, and associated risk factors in the cohort of LTx recipients. In particular, we will determine the impact and interaction of bacteria, viruses and fungi in the transplanted lung, in order to inform improved treatment and prevention of CLAD.

6. STUDY PROPOSAL

LTx saves lives, however the long-term viability of the allograft is limited by the immune response to it and the development of CLAD. The production of *de novo* anti-HLA alloreactive donor-specific antibodies (DSA) is deleterious to the graft and is associated with CLAD. However, the immune cell response following LTx and its relationship to the lung microbiome is poorly understood. We propose a thorough and integrated delineation of the immune response to LTx. We will obtain a comprehensive picture of the lower airway bacterial, viral and fungal communities in LTx recipients, and determine the implications of the lung microbiomeon inflammation, lung tissue remodelling pathways and allograft function. Following LTx, we will have defined immune and physiological biomarkers of rejection that will better inform the use of appropriately focused immunosuppressive therapies with a view to reducing CLAD and prolonging survival following LTx. Insights gained will extend beyond LTx to all types of solid organ transplantation.

7. RESEARCH PLAN

Specific Aim #1: Defining HLA eplet mismatch scores

Rationale: The humoral antibody-antigen immune response describes the interaction between anti-HLA DSA with the cognate non-self HLA molecule expressed within the lung allograft. HLA genes are highly polymorphic with over 10,000 HLA Class I and II alleles. Anti-HLA antibodies recognise specific exposed regions of the HLA antigen that consist of chains of amino acid sequence located within the complement determining regions, termed eplets.. Approximately 600 HLA Class I and II eplets have been described by Duquesnoy *et al.* (10) using the theoretical computer algorithm HLAMatchmaker. Functional eplets specifically recognise HLA antibodies and are exposed within the binding groove demonstrating

antigenicity, i.e. reactivity with antibody, and immunogenicity, i.e. ability to elicit immune response. LTx recipients are transplanted with HLA-mismatched donor lungs.

HLA typing will be performed by Luminex sequence-specific oligonucleotide or sequenced-based typing for HLA-A, -B, -C, -DR and -DQ (Victorian Transplantation and Immunogenetics Service). The HLAMatchmaker 500 pair (ABC and DRDQ eplet) program will be used as a research tool to assess eplet matching for all LTx (http://www.hlamatchmaker.net/). The calculated linear score of structural diversity for each LTx will be used for the subsequent studies looking at the impact of structural incompatibility on immune pathways and allograft dysfunction.

Specific Aim #2: Immune response in high eplet mismatch lung transplant recipients

i) De novo DSA production

Rationale: Anti-HLA DSA may be present at the time of LTx (sensitised recipient) or develop *de novo* following LTx, and may be specific to HLA-class I and/or HLA Class II. The alloreactivity of DSA is determined by whether they are bound within the lung allograft, and whether they amplify an immune response. The C1q assay is a modification of the standard Luminex assay that distinguishes complement-fixing (and therefore injurious) from non-complement-fixing DSA. The clinical utility of the C1q assay has been demonstrated in renal transplantation, and similar studies are awaited in LTx. DSA can also be alloreactive via complement-independent pathways. NK cells and macrophages can be directly activated via the low-affinity Fc receptor resulting in antibody-independent cell-mediated cytotoxicity. Aim #2 will integrate DSA results with eplet mismatch scores and other immune markers to provide a comprehensive description of Antibody Mediated Rejection (AMR).

ii) B-cell specificity, phenotype and function

Rationale: B cells can be alloreactive, either as precursors (naïve or memory B cells) of DSA-producing plasma cells or as antigen-presenting cells to allospecific T-cells. HLA tetramers provide an opportunity to track antigen-specific B cell responses longitudinally from the time of LTx. In LTx, the dynamics and role of alloreactive B cells in producing anti-HLA DSA and graft dysfunction has not been described.

B cell subtypes will be phenotyped from the blood of LTx recipients by flow cytometry in the lab of Prof Function of immune cells will be assessed using a stimulated intracellular cytokine stain. The dynamics of B and T cell biology will be tracked longitudinally over the first 12

months, and will be correlated with eplet mismatch scores and *de novo* DSA production (assessed at 3 and 12 months post-transplant).

iii) Antibody-dependent NK cell cytotoxicity

Rationale: The complement-independent mechanisms that lead to AMR are poorly understood, although recent work suggests that NK cells may be important effectors of Antibody-Dependent Cell-Mediated Cytoxicity (ADCC). We have previously shown that NK cells are activated in LTx recipients and contribute to DSA-mediated allograft injury (5). Molecular signatures from allograft biopsies support the alloreactive potential of NK cells. We aim to use an *in vitro* NK-cell ADCC assay to analyse the role of DSAs in promoting NK cell activation. NK cells will be identified by flow cytometry (CD3- CD56+ lymphocytes) in the blood LTx patients at 3 and 12 months post-transplant.

Specific Aim #3: Determine the immunological profiles of the lower airways of lung transplant recipients

We have developed approaches utilizing multiplexed quantitative polymerase chain reactions (PCRs) to determine BAL cell (predominantly macrophage) gene expression in transplant recipients, which allowed individuals to be clustered based upon the immunological profile of their lower airways. We will now utilize our gene expression profiling toolkit in order to define the immunological micro-environment present in the LTx recipients at each timepoint (i.e. 2 weeks, 3, 6, 9, 12 and 18 months). The gene expression dataset we will generate, which will be integrated with the clinical metadata, will be the initial point of comparison with the microbial datasets to be generated in the following sections.

Specific Aim #4: Identification of the bacterial, fungal and viral communities in lung transplant recipients

Bacterial and fungal community identification: Fungal and bacterial DNA will be amplified and sequenced. The virome will be characterised, using two experimental approaches in order to ensure we capture a complete and informative dataset. First, PCR amplification will be used to analyse the total set of BAL samples informing on the carriage of a specific set of RNA and DNA viruses, including a panel of anelloviruses which we hypothesize will be highly relevant in the lung transplant setting. In a second step, we will refine the patient pool for analysis based upon the clinical, immunological, bacterial, fungal and viral PCR results and perform metagenomic sequencing providing us with in-depth unbiased data on viral carriage.

Specific Aim #5: Bioinformatics and integration of microbial, immunological and clinical datasets

Network-based approaches have emerged as a powerful way to study microbial systems and analyse high-dimensional datasets. Using the bacterial, fungal and viral sequencing datasets, we will construct and combine ecological networks using dissimilarity and correlation-based methods. This will firstly allow us to determine the extent to which bacterial, fungal and viral communities are connected by causal relationships, and in particular, we can identify "key-stone" species which would be predicted to play a major role in community dynamics and consequently the immunological and health status of the transplant recipient.

8. METHODOLOGY

8.1 Patient Recruitment:

All adult patients undergoing lung transplantation, and whose long-term follow-up will be at the Alfred Hospital will be invited to participate in the study. Patients would be enrolled prior to transplantation.

8.2 Sample Collection:

Blood and BAL research samples will be collected throughout the project at time points and quantities described here.

Pre-Transplant:

At the time of transplant an additional 20mls of blood will be collected providing a research sample, this will be collected as the same time as the patients routine pre-operative bloods are being taken, not requiring additional intervention.

At Bronchoscopies:

a) As part of routine post-transplant management lung transplant recipients attend for surveillance bronchoscopy at 2 and 6 weeks and 3, 6, 9, 12 and 18 months post-transplant, at which point blood and BAL samples are collected.

b) On occasions of clinical concern, additional bronchoscopies may be scheduled as part of patient care. At these clinical relevant bronchoscopies, blood and BAL samples are also collected.

At both the scheduled surveillance bronchoscopies and the clinically initiated bronchoscopies occurring within the project follow-up period an additional 20mls of blood and 30mls of BAL samples will be collected for this project at the same time as the routine clinical samples are collected.

8.3 Participant Involvement:

All participants will be followed for a total of 3 years following lung transplant

Lung transplant recipients enrolled in the study would not need to make any additional trips to the Alfred hospital nor undertake any additional investigations. Collection of research related samples at bronchoscopies will coincide with routine blood and BAL collections and will not require additional procedures for the participants.

8.4 Sample Processing:

At bronchoscopy, blood and BAL samples will be collected according to standard clinical protocols and forwarded to microbiology for routine diagnostic assessments. These microbiological results will be analysed as an outcome variable in the current study.

Remaining blood and BAL samples will be processed and centrifuged at the Alfred hospital to provide plasma and BAL supernatant samples for biomarker analysis. These samples will then be transported to our collaborators for analysis.

9. INCLUSION/EXCLUSION CRITERIA

Approximately 100 patients will be enrolled at The Alfred Hospital

Inclusion Criteria

- Adults ≥18 years of age
- Lung Transplant Recipients
- Provide Written Consent

Exclusion Criteria

- Long Term Follow-Up not planned for The Alfred Hospital

10. ANALYSIS:

Descriptive statistics will be used to determine the baseline and clinical characteristics of the subjects in the study. Univariate analysis of the biomarkers and their association with the clinical end-points of acute rejection and CLAD will be performed using the Chi-square test for equal proportion, Student's t test for normally distributed continuous variables and the Mann-Whitney U test for non-normally distributed variables. Time to acute rejection and CLAD will be analyzed using univariate and multivariate Cox proportional hazards regression, adjusting for potential confounding factors and reported using the Kaplan Meier curves. All analysis will be performed using SAS version 9.2 (SAS Institute Inc., Cary, NC, USA). A two-sided p-value of 0.05 will be considered to be statistically significant.

11. REFERENCES

1. Paraskeva M et al. (2013) Acute fibrinoid organizing pneumonia after lung transplantation. *Am J Respir Crit Care Med* 187, 1360-1368.

2. Westall G et al. (2017) Activin Biology after Lung Transplantation. *Transplant Direct* 11; 3:e159.

3. Snell G et al. (2015) The potential role of activin and follistatin in lung transplant dysfunction. *Expert Rev Respir Med* 9:697

4. Meehan AC et al. (2013) The impact of commonly used transplant immunosuppressive drugs on human NK cell function is dependent upon stimulation conditions. *PLoS One* 8:e56042

5. Meehan Ac et al. (2010) Natural killer cell activation in the lung allograft early posttransplantation. *Transplantation* 89:756

6. Sullivan LC et al (2015) The presence of HLA-restricted, CMV-specific CD8+ T cells in the blood of human lung transplant recipients correlates with chronic allograft rejection. *PLos One* 10:e135972

7. Walton D et al (2016) HLA matching at the eplet level protects against chronic lung allograft dysfunction. *Am J Transpl* 16:2695

8. Paraskeva M et al. (2011) CMV replication within the lung allograft is associated with bronchiolitis obliterans syndrome. *Am J Transpl* 11: 2190.

9. Jeong W et al (2017). Clinical effectiveness of early posaconazole suspension pre-emptive therapy in lung transplant recipients. Antimicrob Chemother epub ahead of print

10. Duquesnoy RJ (2016) Reflections on HLA epitope based matching for transplantation. Front Immunol 7:469

Alfred Ethics Project Number: 430 / 17

IDENTIFYING IMMUNE AND INFECTIOUS BIOMARKERS OF ALLOGRAFT DYSFUNCTION FOLLOWING LUNG TRANSPLANTATION

Protocol Date: **18th December 2017**

Version Number: **2.0**

Principal Investigator: A/Prof Glen Westall

Co-Investigators: Prof Greg Snell (Alfred Health), Dr Miranda Paraskeva (Alfred Health), Dr Helen Whitford (Alfred Health), Dr Kovi Levin (Alfred Health), Dr Sakhee Kotecha (Alfred Health), Prof Trevor Williams (Alfred Health), A/Prof Bronwyn Levvey (Alfred Health), Prof David Tarlinton (Monash University), Prof Ben Marsland (Monash University), Prof Anton Peleg (Alfred Health), Dr Orla Morrissey (Alfred Health), Dr Lucy Sullivan (Alfred Health and University of Melbourne)

CONTENTS:

1.	Abbreviations	2
2.	Summary	3
3.	Hypothesis	4
4.	Broad Project Aims	4
5.	Background	4
6.	Study Proposal	6
7.	Research Plan	7
8.	Methodology	9
9.	Inclusion/Exclusion Criteria	11
10.	Analysis	11
12.	References	12

12. ABBREVIATIONS

ADCC	Antibody-Dependent Cell-Mediated Cytotoxicity
AFOP	Acute Fibrinoid Organizing Pneumonia
AMR	Antibody Mediated Rejection
BAL	Broncholaveolar Lavage
BOS	Bronchiolitis Obliterans Syndrome
CLAD	Chronic Lung Allograft Dysfunction
DAD	Diffuse Alveolar Damage
DSA	Donor Specific Antibodies
HLA	Human Leukocyte Antigens
LTx	Lung Transplant
MFI	Mean Fluorescence Intensity
MHC	Major Histocompatability Complex
NK	Natural Killer
OB	Obliterative Bronchiolitis
PCRs	Polymerase Chain Reactions
RAS	Restrictive Allograft Syndrome
Tfh	Follicular Helper T Cells

13. SUMMARY

Transplantation saves the lives of patients with end stage lung disease. Better operative techniques, anaesthesia, organ preservation and more potent immunosuppression have improved outcomes significantly but long-term graft survival remains unchanged. Transplanted lungs are lost due to an ill-defined process called chronic lung allograft dysfunction (CLAD) that is characterized by either fibrosis of the airways (bronchiolitis obliterans syndrome = BOS) or of the lung architecture (restrictive allograft syndrome = RAS) (1). CLAD is characterised by a drop in lung function, that once established is irreversible. The major risk factors for CLAD are i) acute rejection as diagnosed on lung biopsy and ii) infection, particularly CMV and aspergillus. To date, biomarkers have not been identified that can diagnose acute rejection or predict the early onset of CLAD.

We have previously explored a number of biomarkers and demonstrated an association with allograft dysfunction following lung transplant (LTx). We have shown that activin and follistatin levels vary with time from LTx and reflect a proinflammatory environment (2,3). We continue to explore the role of NK cells following LTx having previously shown that NK cells become activated in patients undergoing acute rejection or CMV infection (4, 5). Collaborative research with the Doherty Institute has demonstrated a link between immune cells that control CMV and the later development of CLAD (6). We now wish to extend this biomarker approach with a new prospective cohort of LTx patients who will provide blood and BAL samples to allow further interrogation of immune and infectious links to allograft dysfunction following LTx.

Identifying, CLAD at an early stage, may allow therapeutic interventions to interrupt the progression to irreversible damage to the transplanted lung. We propose to analyse in both a cross-sectional and longitudinal study whether a series of biomarkers can predict for episodes of acute rejection or the development of CLAD in a cohort of patients undergoing LTx at the Alfred Hospital.

14. HYPOTHESIS

The identification of biomarkers of immune function and infection in the blood and lung will predict episodes of acute rejection and the onset of chronic lung allograft dysfunction (CLAD) following LTx.

15. BROAD PROJECT AIMS

4.1. To establish the immune response in the serum and broncholaveolar lavage (BAL) as measured cross-sectionally and longitudinally during the three year post-LTx and the association with later episodes of acute rejection or the subsequent development of CLAD.

4.2. To determine whether episodes of viral, fungal or bacterial infection in the LTx recipient trigger further increases in the secretion of these biomarkers and thus act as an inflammatory stimulus.

16. BACKGROUND

5.1 The immune response to lung transplantation

Successful outcomes following LTx depend on our ability to pharmacologically manipulate the immune system to prevent rejection of the lung allograft. The antigens involved in graft rejection are encoded by the major histocompatability complex (MHC). This group of highly polymorphic genes defines the human leukocyte antigens (HLA) which are expressed on virtually all cell surfaces. The polymorphic nature of these allopeptides defines the genetic signature of each individual and allows the host immune system to differentiate between self and foreign peptides. The majority of transplanted lung allografts are MHC-mismatched to the recipient. As such, the transplanted lung will be seen as foreign to the recipient's immune system. This leads to activation of immune pathways that if left unchecked would lead to rejection and destruction of the newly transplanted lung. We are interested in whether structural differences between transplant recipients and their donors, termed eplets, may drive the acute cellular and humoral rejection responses following LTx.

5.2 Chronic Lung Allograft Dysfunction

LTx chronic rejection has been described pathologically as Obliterative Bronchiolitis (OB) and physiologically as airflow limitation, in the form of the Bronchiolitis Obliterans Syndrome (BOS). However, increasingly other phenotypes of chronic rejection have been recognized, in particular the Restrictive Allograft Syndrome (RAS), characterized by restrictive physiology, interstitial infiltrates on radiology, and interstitial fibrosis, acute fibrinoid organizing pneumonia (AFOP) and diffuse alveolar damage (DAD) on histology (REF). To encompass these important emerging variants and concepts of chronic dysfunction, the term Chronic Lung Allograft Dysfunction (CLAD) has recently been created. We have been interested to see whether structural differences between transplant recipients and their donors may drive immune responses that lead to the later development of CLAD. Historically, the degree of matching between a transplant recipient and donor has been determined by counting the number of mismatched HLA-A, B and DR antigens of the donor. HLA antigens have multiple epitopes that can be recognised by specific antibodies. Elucidation of the three-dimensional molecular structures and amino acid sequence differences between HLA antigens allows structural characterisation of HLA antigens, and what are termed eplets. We have recently demonstrated that HLA matching at the eplet level protects against CLAD (7).

5.3 Acute Rejection

Despite the use of maintenance immunosuppression, acute cellular rejection is a common problem post lung transplant with 34% of adult LTx recipients experiencing at least one episode during the first year post-transplant. The lung allograft is particularly susceptible to alloreactivity with the incidence of acute rejection being higher following LTx compared to other solid organ transplants. Whilst the direct effects of acute rejection are usually controlled and reversed following high-dose intravenous steroid treatment, the indirect effects remain problematic, with episodes of acute rejection being strongly associated with the later development of chronic lung allograft dysfunction (CLAD). Given the need for invasive transbronchial biopsies to provide a histological diagnosis of acute cellular rejection, we have previously evaluated biomarkers in the blood and BAL and their association with acute rejection (2-5). Functional assays of T cell and B cell function in the blood may identify patients at higher risk of acute cellular rejection. Analysis of the BAL is very useful in providing mechanistic insights into the immunology of graft function and rejection following LTx. However, BAL findings seen in acute rejection must be interpreted in the context of those described in stable lung transplant recipients, and after taking into account the confounding nature of chronic airway infection.

5.4 Infection in immunosuppressed lung transplant recipients

Infection is commonly encountered following LTx and is largely related to the use of immunosuppressive medication. We have a long-standing interest in how opportunistic infections such as CMV (8) and Aspergillus (9) have both direct effects on the lung allograft, as well as indirect effects through an association with CLAD. The airways, historically thought to be sterile, are now known to harbor a microbiota that changes in its constituents depending upon the health status of an individual. This lung microbiome consists of bacteria, viruses and fungi, which are in intimate contact with cells in the lung providing tonic stimuli to our immune system. As is routine clinical practice, the current study will also include a detailed prospective analysis of infections, and associated risk factors in the cohort of LTx recipients. In particular, we will determine the impact and interaction of bacteria, viruses and fungi in the transplanted lung, in order to inform improved treatment and prevention of CLAD.

17. STUDY PROPOSAL

LTx saves lives, however the long-term viability of the allograft is limited by the immune response to it and the development of CLAD. The production of *de novo* anti-HLA alloreactive donor-specific antibodies (DSA) is deleterious to the graft and is associated with CLAD. However, the immune cell response following LTx and its relationship to the lung microbiome is poorly understood. We propose a thorough and integrated delineation of the immune response to LTx. We will obtain a comprehensive picture of the lower airway bacterial, viral and fungal communities in LTx recipients, and determine the implications of the lung microbiomeon inflammation, lung tissue remodelling pathways and

allograft function. Following LTx, we will have defined immune and physiological biomarkers of rejection that will better inform the use of appropriately focused immunosuppressive therapies with a view to reducing CLAD and prolonging survival following LTx. Insights gained will extend beyond LTx to all types of solid organ transplantation.

18. RESEARCH PLAN

Specific Aim #1: Defining HLA eplet mismatch scores

Rationale: The humoral antibody-antigen immune response describes the interaction between anti-HLA DSA with the cognate non-self HLA molecule expressed within the lung allograft. HLA genes are highly polymorphic with over 10,000 HLA Class I and II alleles. Anti-HLA antibodies recognise specific exposed regions of the HLA antigen that consist of chains of amino acid sequence located within the complement determining regions, termed eplets.. Approximately 600 HLA Class I and II eplets have been described by Duquesnoy *et al.* (10) using the theoretical computer algorithm HLAMatchmaker. Functional eplets specifically recognise HLA antibodies and are exposed within the binding groove demonstrating antigenicity, i.e. reactivity with antibody, and immunogenicity, i.e. ability to elicit immune response. LTx recipients are transplanted with HLA-mismatched donor lungs. HLA typing will be performed by Luminex sequence-specific oligonucleotide or sequenced-based typing for HLA-A, -B, -C, -DR and -DQ (Victorian Transplantation and Immunogenetics Service). The HLAMatchmaker 500 pair (ABC and DRDQ eplet) program will be used as a research tool to assess eplet matching for all LTx (http://www.hlamatchmaker.net/). The calculated linear score of structural diversity for each LTx will be used for the subsequent studies looking at the impact of structural incompatibility on immune pathways and allograft dysfunction.

Specific Aim #2: Immune response in high eplet mismatch lung transplant recipients

iv) De novo DSA production

Rationale: Anti-HLA DSA may be present at the time of LTx (sensitised recipient) or develop *de novo* following LTx, and may be specific to HLA-class I and/or HLA Class II. The alloreactivity of DSA is determined by whether they are bound within the lung allograft, and whether they amplify an immune response. The C1q assay is a modification of the standard Luminex assay that distinguishes complement-fixing (and therefore injurious) from non-complement-fixing DSA. The clinical utility of the C1q assay has been demonstrated in renal transplantation, and similar studies are awaited in LTx. DSA can also be alloreactive via complement-independent pathways. NK cells and macrophages can be directly activated via the low-affinity Fc receptor resulting in antibody-independent cell-mediated

cytotoxicity. Aim #2 will integrate DSA results with eplet mismatch scores and other immune markers to provide a comprehensive description of Antibody Mediated Rejection (AMR).

v) B-cell specificity, phenotype and function

Rationale: B cells can be alloreactive, either as precursors (naïve or memory B cells) of DSA-producing plasma cells or as antigen-presenting cells to allospecific T-cells. HLA tetramers provide an opportunity to track antigen-specific B cell responses longitudinally from the time of LTx. In LTx, the dynamics and role of alloreactive B cells in producing anti-HLA DSA and graft dysfunction has not been described.

B cell subtypes will be phenotyped from the blood of LTx recipients by flow cytometry in the lab of Prof Function of immune cells will be assessed using a stimulated intracellular cytokine stain. The dynamics of B and T cell biology will be tracked longitudinally over the first 12 months, and will be correlated with eplet mismatch scores and *de novo* DSA production (assessed at 3 and 12 months post-transplant).

vi) Antibody-dependent NK cell cytotoxicity

Rationale: The complement-independent mechanisms that lead to AMR are poorly understood, although recent work suggests that NK cells may be important effectors of Antibody-Dependent Cell-Mediated Cytoxicity (ADCC). We have previously shown that NK cells are activated in LTx recipients and contribute to DSA-mediated allograft injury (5). Molecular signatures from allograft biopsies support the alloreactive potential of NK cells. We aim to use an *in vitro* NK-cell ADCC assay to analyse the role of DSAs in promoting NK cell activation. NK cells will be identified by flow cytometry (CD3-CD56+ lymphocytes) in the blood LTx patients at 3 and 12 months post-transplant.

Specific Aim #3: Determine the immunological profiles of the lower airways of lung transplant recipients

We have developed approaches utilizing multiplexed quantitative polymerase chain reactions (PCRs) to determine BAL cell (predominantly macrophage) gene expression in transplant recipients, which allowed individuals to be clustered based upon the immunological profile of their lower airways We will now utilize our gene expression profiling toolkit in order to define the immunological micro-environment present in the LTx recipients at each timepoint (i.e. 2 weeks, 3, 6, 9, 12 and 18 months). The gene expression dataset we will generate, which will be integrated with the clinical metadata, will be the initial point of comparison with the microbial datasets to be generated in the following sections. Representative samples from the patient clusters identified by the multiplexed PCRs will be analysed further by RNAseq in order to gain deeper insight into the immunological profiles of the patient groups.

Specific Aim #4: Identification of the bacterial, fungal and viral communities in lung transplant recipients

Bacterial and fungal community identification: Fungal and bacterial DNA will be amplified and sequenced. The virome will be characterised, using two experimental approaches in order to ensure we capture a complete and informative dataset. First, PCR amplification will be used to analyse the total set of BAL samples informing on the carriage of a specific set of RNA and DNA viruses, including a panel of anelloviruses which we hypothesize will be highly relevant in the lung transplant setting. In a second step, we will refine the patient pool for analysis based upon the clinical, immunological, bacterial, fungal and viral PCR results and perform metagenomic sequencing providing us with in-depth unbiased data on viral carriage.

Specific Aim #5: Bioinformatics and integration of microbial, immunological and clinical datasets
Network-based approaches have emerged as a powerful way to study microbial systems and analyse high-dimensional datasets. Using the bacterial, fungal and viral sequencing datasets, we will construct and combine ecological networks using dissimilarity and correlation-based methods. This will firstly allow us to determine the extent to which bacterial, fungal and viral communities are connected by causal relationships, and in particular, we can identify "key-stone" species which would be predicted to play a major role in community dynamics and consequently the immunological and health status of the transplant recipient.

Specific Aim #6: Applying microarray to describe the molecular phenotype of lung allografts
The current standard of biopsy-based assessment of lung transplants is the ISHLT histology classification applied to transbronchial biopsies. This assessment is based largely on empirical classifications and expert opinions, and provides little insight into the pathophysiological and immunological drivers of CLAD. Microarray analysis establishes diagnostic patterns of abnormality (and normality), but as yet is not been rigorously applied in the LTx setting.
We will utilise Microarray Analysis at 3 and 12 months post-transplant, scores provided by the Molecular Microscope Diagnostic System used to guide diagnosis of T cell mediated rejection and AMR will be applied to the lung allograft biopsies provided. These Microarray results will be correlated with eplet mismatch scores and immune markers and CLAD.

19. METHODOLOGY

8.1 Patient Recruitment:

All adult patients undergoing lung transplantation, and whose long-term follow-up will be at the Alfred Hospital will be invited to participate in the study. Patients would be enrolled prior to transplantation.

8.2 Sample Collection:

8.2.1 Blood and BAL research samples will be collected throughout the project at time points and quantities described here.

Pre-Transplant:

At the time of transplant an additional 20mls of blood will be collected providing a research sample, this will be collected as the same time as the patients routine pre-operative bloods are being taken, not requiring additional intervention.

At Bronchoscopies:

c) As part of routine post-transplant management lung transplant recipients attend for surveillance bronchoscopy at 2 and 6 weeks and 3, 6, 9, 12 and 18 months post-transplant, at which point blood and BAL samples are collected.
d) On occasions of clinical concern, additional bronchoscopies may be scheduled as part of patient care. At these clinical relevant bronchoscopies, blood and BAL samples are also collected.

At both the scheduled surveillance bronchoscopies and the clinically initiated bronchoscopies occurring within the project follow-up period an additional 20mls of blood and 30mls of BAL samples will be collected for this project at the same time as the routine clinical samples are collected.

8.2.2 Transbronchial Biopsy Samples will be collected throughout the project at the time points described here:

At participant's routine 3 and 12month post-transplant bronchoscopy, two additional transbronchial biopsy bites will be collected and sent to Canada for Mircoarray Analysis.

8.3 Participant Involvement:

All participants will be followed for a total of 3 years following lung transplant.

Lung transplant recipients enrolled in the study would not need to make any additional trips to the Alfred hospital nor undertake any additional investigations. Collection of research related samples at bronchoscopies will coincide with routine biopsy, blood and BAL collections and will not require additional procedures for the participants.

8.4 Sample Processing:

At bronchoscopy, blood and BAL samples will be collected according to standard clinical protocols and forwarded to microbiology for routine diagnostic assessments. These microbiological results will be analysed as an outcome variable in the current study.

Remaining blood and BAL samples will be processed and centrifuged at the Alfred hospital to provide plasma and BAL supernatant samples for biomarker analysis. These samples will then be transported to our collaborators for analysis.

Transbronchial biopsy samples will be collected according to standard clinical protocols; the tissue will be placed into RNAlater and shipped by courier to Alberta, Canada at ambient temperature to Alberta Transplant Applied Genomic Centre (ATAGC) for analysis.

20. INCLUSION/EXCLUSION CRITERIA

Approximately 100 patients will be enrolled at The Alfred Hospital

Inclusion Criteria

- Adults ≥18 years of age
- Lung Transplant Recipients
- Provide Written Consent

Exclusion Criteria

- Long Term Follow-Up not planned for The Alfred Hospital

21. ANALYSIS:

Descriptive statistics will be used to determine the baseline and clinical characteristics of the subjects in the study. Univariate analysis of the biomarkers and their association with the clinical end-points of acute rejection and CLAD will be performed using the Chi-square test for equal proportion, Student's t test for normally distributed continuous variables and the Mann-Whitney U test for non-normally distributed variables. Time to acute rejection and CLAD will be analyzed using univariate and multivariate Cox proportional hazards regression, adjusting for potential confounding factors and reported using the Kaplan Meier curves. All analysis will be performed using SAS version 9.2 (SAS Institute Inc., Cary, NC, USA). A two-sided p-value of 0.05 will be considered to be statistically significant.

22. REFERENCES

11. Paraskeva M et al. (2013) Acute fibrinoid organizing pneumonia after lung transplantation. *Am J Respir Crit Care Med* 187, 1360-1368.

12. Westall G et al. (2017) Activin Biology after Lung Transplantation. *Transplant Direct* 11; 3:e159.

13. Snell G et al. (2015) The potential role of activin and follistatin in lung transplant dysfunction. *Expert Rev Respir Med* 9:697

14. Meehan AC et al. (2013) The impact of commonly used transplant immunosuppressive drugs on human NK cell function is dependent upon stimulation conditions. *PLoS One* 8:e56042

15. Meehan Ac et al. (2010) Natural killer cell activation in the lung allograft early posttransplantation. *Transplantation* 89:756

16. Sullivan LC et al (2015) The presence of HLA-restricted, CMV-specific CD8+ T cells in the blood of human lung transplant recipients correlates with chronic allograft rejection. *PLos One* 10:e135972

17. Walton D et al (2016) HLA matching at the eplet level protects against chronic lung allograft dysfunction. *Am J Transpl* 16:2695

18. Paraskeva M et al. (2011) CMV replication within the lung allograft is associated with bronchiolitis obliterans syndrome. *Am J Transpl* 11: 2190.

19. Jeong W et al (2017). Clinical effectiveness of early posaconazole suspension pre-emptive therapy in lung transplant recipients. Antimicrob Chemother epub ahead of print

20. Duquesnoy RJ (2016) Reflections on HLA epitope based matching for transplantation. Front Immunol 7:469

IDENTIFYING IMMUNE AND INFECTIOUS BIOMARKERS OF ALLOGRAFT DYSFUNCTION FOLLOWING LUNG TRANSPLANTATION

Protocol Date: **19th June 2019**
Version Number: **3.0**

Principal Investigator: A/Prof Glen Westall

Co-Investigators: Prof Greg Snell (Alfred Health), Dr Miranda Paraskeva (Alfred Health), Dr Helen Whitford (Alfred Health), Dr Kovi Levin (Alfred Health), Dr Sakhee Kotecha (Alfred Health), Prof Trevor Williams (Alfred Health), A/Prof Bronwyn Levvey (Alfred Health), Prof David Tarlinton (Monash University), Prof Ben Marsland (Monash University), Prof Anton Peleg (Alfred Health), Dr Orla Morrissey (Alfred Health), Dr Lucy Sullivan (Alfred Health and University of Melbourne)

CONTENTS:

1. Abbreviations 2
2. Summary 3
3. Hypothesis 4
4. Broad Project Aims 4
5. Background 4
6. Study Proposal 6
7. Research Plan 7
8. Methodology 9
9. Inclusion/Exclusion Criteria 11
10. Analysis 11
12. References 12

23. ABBREVIATIONS

ADCC	Antibody-Dependent Cell-Mediated Cytotoxicity
AFOP	Acute Fibrinoid Organizing Pneumonia
AMR	Antibody Mediated Rejection
BAL	Broncholaveolar Lavage
BOS	Bronchiolitis Obliterans Syndrome
CLAD	Chronic Lung Allograft Dysfunction
DAD	Diffuse Alveolar Damage
DSA	Donor Specific Antibodies
HLA	Human Leukocyte Antigens
LTx	Lung Transplant
MFI	Mean Fluorescence Intensity
MHC	Major Histocompatability Complex
NK	Natural Killer
OB	Obliterative Bronchiolitis
PCRs	Polymerase Chain Reactions
RAS	Restrictive Allograft Syndrome
Tfh	Follicular Helper T Cells

24. SUMMARY

Transplantation saves the lives of patients with end stage lung disease. Better operative techniques, anaesthesia, organ preservation and more potent immunosuppression have improved outcomes significantly but long-term graft survival remains unchanged. Transplanted lungs are lost due to an ill-defined process called chronic lung allograft dysfunction (CLAD) that is characterized by either fibrosis of the airways (bronchiolitis obliterans syndrome = BOS) or of the lung architecture (restrictive allograft syndrome = RAS) (1). CLAD is characterised by a drop in lung function, that once established is irreversible. The major risk factors for CLAD are i) acute rejection as diagnosed on lung biopsy and ii) infection, particularly CMV and aspergillus. To date, biomarkers have not been identified that can diagnose acute rejection or predict the early onset of CLAD.

We have previously explored a number of biomarkers and demonstrated an association with allograft dysfunction following lung transplant (LTx). We have shown that activin and follistatin levels vary with time from LTx and reflect a proinflammatory environment (2,3). We continue to explore the role of NK cells following LTx having previously shown that NK cells become activated in patients undergoing acute rejection or CMV infection (4, 5). Collaborative research with the Doherty Institute has demonstrated a link between immune cells that control CMV and the later development of CLAD (6). We now wish to extend this biomarker approach with a new prospective cohort of LTx patients who will provide blood and BAL samples to allow further interrogation of immune and infectious links to allograft dysfunction following LTx.

Identifying, CLAD at an early stage, may allow therapeutic interventions to interrupt the progression to irreversible damage to the transplanted lung. We propose to analyse in both a cross-sectional and longitudinal study whether a series of biomarkers can predict for episodes of acute rejection or the development of CLAD in a cohort of patients undergoing LTx at the Alfred Hospital.

25. HYPOTHESIS

The identification of biomarkers of immune function and infection in the blood and lung will predict episodes of acute rejection and the onset of chronic lung allograft dysfunction (CLAD) following LTx.

26. BROAD PROJECT AIMS

4.1. To establish the immune response in the serum and broncholaveolar lavage (BAL) as measured cross-sectionally and longitudinally during the three year post-LTx and the association with later episodes of acute rejection or the subsequent development of CLAD.

4.2. To determine whether episodes of viral, fungal or bacterial infection in the LTx recipient trigger further increases in the secretion of these biomarkers and thus act as an inflammatory stimulus.

27. BACKGROUND

5.1 The immune response to lung transplantation

Successful outcomes following LTx depend on our ability to pharmacologically manipulate the immune system to prevent rejection of the lung allograft. The antigens involved in graft rejection are encoded by the major histocompatability complex (MHC). This group of highly polymorphic genes defines the human leukocyte antigens (HLA) which are expressed on virtually all cell surfaces. The polymorphic nature of these allopeptides defines the genetic signature of each individual and allows the host immune system to differentiate between self and foreign peptides. The majority of transplanted lung allografts are MHC-mismatched to the recipient. As such, the transplanted lung will be seen as foreign to the recipient's immune system. This leads to activation of immune pathways that if left unchecked would lead to rejection and destruction of the newly transplanted lung. We are interested in whether structural differences between transplant recipients and their donors, termed eplets, may drive the acute cellular and humoral rejection responses following LTx.

5.2 Chronic Lung Allograft Dysfunction

LTx chronic rejection has been described pathologically as Obliterative Bronchiolitis (OB) and physiologically as airflow limitation, in the form of the Bronchiolitis Obliterans Syndrome (BOS). However, increasingly other phenotypes of chronic rejection have been recognized, in particular the Restrictive Allograft Syndrome (RAS), characterized by restrictive physiology, interstitial infiltrates on radiology, and interstitial fibrosis, acute fibrinoid organizing pneumonia (AFOP) and diffuse alveolar damage (DAD) on histology (REF). To encompass these important emerging variants and concepts of chronic dysfunction, the term Chronic Lung Allograft Dysfunction (CLAD) has recently been created. We have been interested to see whether structural differences between transplant recipients and their donors may drive immune responses that lead to the later development of CLAD. Historically, the degree of matching between a transplant recipient and donor has been determined by counting the number of mismatched HLA-A, B and DR antigens of the donor. HLA antigens have multiple epitopes that can be recognised by specific antibodies. Elucidation of the three-dimensional molecular structures and amino acid sequence differences between HLA antigens allows structural characterisation of HLA antigens, and what are termed eplets. We have recently demonstrated that HLA matching at the eplet level protects against CLAD (7).

5.3 Acute Rejection

Despite the use of maintenance immunosuppression, acute cellular rejection is a common problem post lung transplant with 34% of adult LTx recipients experiencing at least one episode during the first year

post-transplant. The lung allograft is particularly susceptible to alloreactivity with the incidence of acute rejection being higher following LTx compared to other solid organ transplants. Whilst the direct effects of acute rejection are usually controlled and reversed following high-dose intravenous steroid treatment, the indirect effects remain problematic, with episodes of acute rejection being strongly associated with the later development of chronic lung allograft dysfunction (CLAD). Given the need for invasive transbronchial biopsies to provide a histological diagnosis of acute cellular rejection, we have previously evaluated biomarkers in the blood and BAL and their association with acute rejection (2-5). Functional assays of T cell and B cell function in the blood may identify patients at higher risk of acute cellular rejection. Analysis of the BAL is very useful in providing mechanistic insights into the immunology of graft function and rejection following LTx. However, BAL findings seen in acute rejection must be interpreted in the context of those described in stable lung transplant recipients, and after taking into account the confounding nature of chronic airway infection.

5.4 Infection in immunosuppressed lung transplant recipients

Infection is commonly encountered following LTx and is largely related to the use of immunosuppressive medication. We have a long-standing interest in how opportunistic infections such as CMV (8) and Aspergillus (9) have both direct effects on the lung allograft, as well as indirect effects through an association with CLAD. The airways, historically thought to be sterile, are now known to harbor a microbiota that changes in its constituents depending upon the health status of an individual. This lung microbiome consists of bacteria, viruses and fungi, which are in intimate contact with cells in the lung providing tonic stimuli to our immune system. As is routine clinical practice, the current study will also include a detailed prospective analysis of infections, and associated risk factors in the cohort of LTx recipients. In particular, we will determine the impact and interaction of bacteria, viruses and fungi in the transplanted lung, in order to inform improved treatment and prevention of CLAD.

28. STUDY PROPOSAL

LTx saves lives, however the long-term viability of the allograft is limited by the immune response to it and the development of CLAD. The production of *de novo* anti-HLA alloreactive donor-specific antibodies (DSA) is deleterious to the graft and is associated with CLAD. However, the immune cell response following LTx and its relationship to the lung microbiome is poorly understood. We propose a thorough and integrated delineation of the immune response to LTx. We will obtain a comprehensive picture of the lower airway bacterial, viral and fungal communities in LTx recipients, and determine the implications of the lung microbiomeon inflammation, lung tissue remodelling pathways and allograft function. Following LTx, we will have defined immune and physiological biomarkers of rejection that will better inform the use of appropriately focused immunosuppressive therapies with a

view to reducing CLAD and prolonging survival following LTx. Insights gained will extend beyond LTx to all types of solid organ transplantation.

29. RESEARCH PLAN

Specific Aim #1: Defining HLA eplet mismatch scores

Rationale: The humoral antibody-antigen immune response describes the interaction between anti-HLA DSA with the cognate non-self HLA molecule expressed within the lung allograft. HLA genes are highly polymorphic with over 10,000 HLA Class I and II alleles. Anti-HLA antibodies recognise specific exposed regions of the HLA antigen that consist of chains of amino acid sequence located within the complement determining regions, termed eplets.. Approximately 600 HLA Class I and II eplets have been described by Duquesnoy *et al.* (10) using the theoretical computer algorithm HLAMatchmaker. Functional eplets specifically recognise HLA antibodies and are exposed within the binding groove demonstrating antigenicity, i.e. reactivity with antibody, and immunogenicity, i.e. ability to elicit immune response. LTx recipients are transplanted with HLA-mismatched donor lungs. HLA typing will be performed by Luminex sequence-specific oligonucleotide or sequenced-based typing for HLA-A, -B, -C, -DR and -DQ (Victorian Transplantation and Immunogenetics Service). The HLAMatchmaker 500 pair (ABC and DRDQ eplet) program will be used as a research tool to assess eplet matching for all LTx (http://www.hlamatchmaker.net/). The calculated linear score of structural diversity for each LTx will be used for the subsequent studies looking at the impact of structural incompatibility on immune pathways and allograft dysfunction.

Specific Aim #2: Immune response in high eplet mismatch lung transplant recipients

vii) De novo DSA production

Rationale: Anti-HLA DSA may be present at the time of LTx (sensitised recipient) or develop *de novo* following LTx, and may be specific to HLA-class I and/or HLA Class II. The alloreactivity of DSA is determined by whether they are bound within the lung allograft, and whether they amplify an immune response. The C1q assay is a modification of the standard Luminex assay that distinguishes complement-fixing (and therefore injurious) from non-complement-fixing DSA. The clinical utility of the C1q assay has been demonstrated in renal transplantation, and similar studies are awaited in LTx. DSA can also be alloreactive via complement-independent pathways. NK cells and macrophages can be directly activated via the low-affinity Fc receptor resulting in antibody-independent cell-mediated cytotoxicity. Aim #2 will integrate DSA results with eplet mismatch scores and other immune markers to provide a comprehensive description of Antibody Mediated Rejection (AMR).

viii) B-cell specificity, phenotype and function

Rationale: B cells can be alloreactive, either as precursors (naïve or memory B cells) of DSA-producing plasma cells or as antigen-presenting cells to allospecific T-cells. HLA tetramers provide an opportunity to track antigen-specific B cell responses longitudinally from the time of LTx. In LTx, the dynamics

and role of alloreactive B cells in producing anti-HLA DSA and graft dysfunction has not been described.

B cell subtypes will be phenotyped from the blood of LTx recipients by flow cytometry in the lab of Prof Function of immune cells will be assessed using a stimulated intracellular cytokine stain. The dynamics of B and T cell biology will be tracked longitudinally over the first 12 months, and will be correlated with eplet mismatch scores and *de novo* DSA production (assessed at 3 and 12 months post-transplant).

ix) Antibody-dependent NK cell cytotoxicity

Rationale: The complement-independent mechanisms that lead to AMR are poorly understood, although recent work suggests that NK cells may be important effectors of Antibody-Dependent Cell-Mediated Cytoxicity (ADCC). We have previously shown that NK cells are activated in LTx recipients and contribute to DSA-mediated allograft injury (5). Molecular signatures from allograft biopsies support the alloreactive potential of NK cells. We aim to use an *in vitro* NK-cell ADCC assay to analyse the role of DSAs in promoting NK cell activation. NK cells will be identified by flow cytometry (CD3-CD56+ lymphocytes) in the blood LTx patients at 3 and 12 months post-transplant.

Specific Aim #3: Determine the immunological profiles of the lower airways of lung transplant recipients

We will characterise the immunological environment of the lower airways of LTx recipients at each timepoint (i.e. 2 weeks, 3, 6, 9, 12 and 18 months) by RNAseq of immune cells isolated from the lavage fluid. We will perform Mass Spectrometry of the lavage fluid to characterise the metabolome, lipidome and proteome. Bioinformatic integration of these datasets (see Aim 5) will allow us to gain deep insight into the immunological profiles of the patient groups.

Specific Aim #4: Identification of the bacterial, fungal and viral communities in lung transplant recipients

Bacterial and fungal community identification: Fungal and bacterial DNA will be amplified and sequenced. The virome will be characterised, using two experimental approaches in order to ensure we capture a complete and informative dataset. First, PCR amplification will be used to analyse the total set of BAL samples informing on the carriage of a specific set of RNA and DNA viruses, including a panel of anelloviruses which we hypothesize will be highly relevant in the lung transplant setting. In a second step, we will refine the patient pool for analysis based upon the clinical, immunological, bacterial, fungal and viral PCR results and perform metagenomic sequencing providing us with in-depth unbiased data on viral carriage.

Specific Aim #5: Bioinformatics and integration of microbial, immunological and clinical datasets

Network-based approaches have emerged as a powerful way to study microbial systems and analyse high-dimensional datasets. Using the bacterial, fungal and viral sequencing datasets, we will construct and combine ecological networks using dissimilarity and correlation-based methods. This will firstly allow us to determine the extent to which bacterial, fungal and viral communities are connected by causal relationships, and in particular, we can identify "key-stone" species which would be predicted to play a major role in community dynamics and consequently the immunological and health status of the transplant recipient.

Specific Aim #6: Applying microarray to describe the molecular phenotype of lung allografts

The current standard of biopsy-based assessment of lung transplants is the ISHLT histology classification applied to transbronchial biopsies. This assessment is based largely on empirical classifications and expert opinions, and provides little insight into the pathophysiological and immunological drivers of CLAD. Microarray analysis establishes diagnostic patterns of abnormality (and normality), but as yet is not been rigorously applied in the LTx setting.

We will utilise Microarray Analysis at 3 and 12 months post-transplant, scores provided by the Molecular Microscope Diagnostic System used to guide diagnosis of T cell mediated rejection and AMR will be applied to the lung allograft biopsies provided. These Microarray results will be correlated with eplet mismatch scores and immune markers and CLAD.

SUB-STUDY: Single Lung Transplant (SLTx) Recipients

In this sub-study we will perform Specific Aims 3-5 of the main study, above, on the lavage fluid from both the transplanted lobe and the native lobe. The datasets generated from the different lobes will be compared in order to ascertain whether the immunological and microbiological characteristics of the transplanted lobe develops independently, or in parallel, with the native lobes. This analysis will inform whether the local microenvironmental changes within individual lobes influence the health trajectory of transplant recipients.

30. METHODOLOGY

8.1 Patient Recruitment:

All adult patients undergoing lung transplantation, and whose long-term follow-up will be at the Alfred Hospital will be invited to participate in the study. Patients would be enrolled prior to transplantation.

SLTx Sub-Study: Patients who are awaiting or receive a single lung transplant (SLTx) at the Alfred Hospital will be invited to take part in a sub study comparing their transplanted lung with their own

native (non-transplant) lung. These patients will be consented to the main study prior to transplant, and will be asked to sign an addendum of consent if they agree to take part in the sub study.

8.2 Sample Collection:

8.2.1 Blood and BAL research samples will be collected throughout the project at time points and quantities described here.

Pre-Transplant:

At the time of transplant an additional 20mls of blood will be collected providing a research sample, this will be collected as the same time as the patients routine pre-operative bloods are being taken, not requiring additional intervention.

At Bronchoscopies:

e) As part of routine post-transplant management lung transplant recipients attend for surveillance bronchoscopy at 2 and 6 weeks and 3, 6, 9, 12 and 18 months post-transplant, at which point blood and BAL samples are collected.
f) On occasions of clinical concern, additional bronchoscopies may be scheduled as part of patient care. At these clinical relevant bronchoscopies, blood and BAL samples are also collected.

At both the scheduled surveillance bronchoscopies and the clinically initiated bronchoscopies occurring within the project follow-up period an additional 20mls of blood and 30mls of BAL samples will be collected for this project at the same time as the routine clinical samples are collected.

8.2.2 Transbronchial Biopsy Samples will be collected throughout the project at the time points described here:

At participant's routine 3 and 12month post-transplant bronchoscopy, two additional transbronchial biopsy bites will be collected and sent to Canada for Mircoarray Analysis.

8.2.3 SLTx Sub-Study Sample Collection:

At Bronchoscopies:

a) As part of routine post-transplant management, all lung transplant recipients undergo a surveillance bronchoscopy day 1 post lung transplant. For participants who have consented

to take part in the SLTx Sub Study prior to transplant, 30mls of BAL sample will be collected during this bronchoscopy from both the recipient's non-transplant and transplanted lung.

b) During Bronchoscopies as per 8.2.1, in addition to the sample collected as part of the project, participants who consent to take part in the SLTX Sub Study will have 30mls of BAL collected from their non-transplant lung.

8.3 Participant Involvement:

All participants will be followed for a total of 3 years following lung transplant.

Lung transplant recipients enrolled in the study would not need to make any additional trips to the Alfred hospital nor undertake any additional investigations. Collection of research related samples at bronchoscopies will coincide with routine biopsy, blood and BAL collections and will not require additional procedures for the participants.

8.4 Sample Processing:

At bronchoscopy, blood and BAL samples will be collected according to standard clinical protocols and forwarded to microbiology for routine diagnostic assessments. These microbiological results will be analysed as an outcome variable in the current study.

Remaining blood and BAL samples will be processed and centrifuged at the Alfred hospital to provide plasma and BAL supernatant samples for biomarker analysis. These samples will then be transported to our collaborators for analysis.

Transbronchial biopsy samples will be collected according to standard clinical protocols; the tissue will be placed into RNAlater and shipped by courier to Alberta, Canada at ambient temperature to Alberta Transplant Applied Genomic Centre (ATAGC) for analysis.

31. INCLUSION/EXCLUSION CRITERIA

Approximately 150 patients will be enrolled at The Alfred Hospital

Inclusion Criteria

- Adults ≥18 years of age
- Lung Transplant Recipients
- Provide Written Consent

Exclusion Criteria

- Long Term Follow-Up not planned for The Alfred Hospital

32. ANALYSIS:

Descriptive statistics will be used to determine the baseline and clinical characteristics of the subjects in the study. Univariate analysis of the biomarkers and their association with the clinical end-points of acute rejection and CLAD will be performed using the Chi-square test for equal proportion, Student's t test for normally distributed continuous variables and the Mann-Whitney U test for non-normally distributed variables. Time to acute rejection and CLAD will be analyzed using univariate and multivariate Cox proportional hazards regression, adjusting for potential confounding factors and reported using the Kaplan Meier curves. All analysis will be performed using SAS version 9.2 (SAS Institute Inc., Cary, NC, USA). A two-sided p-value of 0.05 will be considered to be statistically significant.

33. REFERENCES

21. Paraskeva M et al. (2013) Acute fibrinoid organizing pneumonia after lung transplantation. *Am J Respir Crit Care Med* 187, 1360-1368.

22. Westall G et al. (2017) Activin Biology after Lung Transplantation. *Transplant Direct* 11; 3:e159.

23. Snell G et al. (2015) The potential role of activin and follistatin in lung transplant dysfunction. *Expert Rev Respir Med* 9:697

24. Meehan AC et al. (2013) The impact of commonly used transplant immunosuppressive drugs on human NK cell function is dependent upon stimulation conditions. *PLoS One* 8:e56042

25. Meehan Ac et al. (2010) Natural killer cell activation in the lung allograft early posttransplantation. *Transplantation* 89:756

26. Sullivan LC et al (2015) The presence of HLA-restricted, CMV-specific CD8+ T cells in the blood of human lung transplant recipients correlates with chronic allograft rejection. *PLos One* 10:e135972

27. Walton D et al (2016) HLA matching at the eplet level protects against chronic lung allograft dysfunction. *Am J Transpl* 16:2695

28. Paraskeva M et al. (2011) CMV replication within the lung allograft is associated with bronchiolitis obliterans syndrome. *Am J Transpl* 11: 2190.

29. Jeong W et al (2017). Clinical effectiveness of early posaconazole suspension pre-emptive therapy in lung transplant recipients. Antimicrob Chemother epub ahead of print

30. Duquesnoy RJ (2016) Reflections on HLA epitope based matching for transplantation. Front Immunol 7:469

Alfred Ethics Project Number: 430 / 17

IDENTIFYING IMMUNE AND INFECTIOUS BIOMARKERS OF ALLOGRAFT DYSFUNCTION FOLLOWING LUNG TRANSPLANTATION

Protocol Date: **16th February 2021**
Version Number: **4.0**

Principal Investigator: Professor Glen Westall

Co-Investigators: Prof Greg Snell (Alfred Health), Dr Miranda Paraskeva (Alfred Health), Dr Helen Whitford (Alfred Health), Dr Kovi Levin (Alfred Health), Dr Sakhee Kotecha (Alfred Health), Prof Trevor Williams (Alfred Health), A/Prof Bronwyn Levvey (Alfred Health), Prof David Tarlinton (Monash University), Prof Ben Marsland (Monash University), Prof Anton Peleg (Alfred Health), Dr Orla Morrissey (Alfred Health), Dr Lucy Sullivan (Alfred Health and University of Melbourne)

CONTENTS:

1.	Abbreviations	2
2.	Summary	3
3.	Hypothesis	4
4.	Broad Project Aims	4
5.	Background	4
6.	Study Proposal	6
7.	Research Plan	7
8.	Methodology	9
9.	Inclusion/Exclusion Criteria	11
10.	Analysis	11
12.	References	12

34. ABBREVIATIONS

ADCC	Antibody-Dependent Cell-Mediated Cytotoxicity
AFOP	Acute Fibrinoid Organizing Pneumonia
AMR	Antibody Mediated Rejection
BAL	Broncholaveolar Lavage
BOS	Bronchiolitis Obliterans Syndrome
CLAD	Chronic Lung Allograft Dysfunction
DAD	Diffuse Alveolar Damage
DSA	Donor Specific Antibodies
HLA	Human Leukocyte Antigens
LTx	Lung Transplant
MFI	Mean Fluorescence Intensity
MHC	Major Histocompatability Complex
NK	Natural Killer
OB	Obliterative Bronchiolitis
PCRs	Polymerase Chain Reactions
RAS	Restrictive Allograft Syndrome
Tfh	Follicular Helper T Cells

35. SUMMARY

Transplantation saves the lives of patients with end stage lung disease. Better operative techniques, anaesthesia, organ preservation and more potent immunosuppression have improved outcomes significantly but long-term graft survival remains unchanged. Transplanted lungs are lost due to an ill-defined process called chronic lung allograft dysfunction (CLAD) that is characterized by either fibrosis of the airways (bronchiolitis obliterans syndrome = BOS) or of the lung architecture (restrictive allograft syndrome = RAS) (1). CLAD is characterised by a drop in lung function, that once established is irreversible. The major risk factors for CLAD are i) acute rejection as diagnosed on lung biopsy and ii) infection, particularly CMV and aspergillus. To date, biomarkers have not been identified that can diagnose acute rejection or predict the early onset of CLAD.

We have previously explored a number of biomarkers and demonstrated an association with allograft dysfunction following lung transplant (LTx). We have shown that activin and follistatin levels vary with time from LTx and reflect a proinflammatory environment (2,3). We continue to explore the role of NK cells following LTx having previously shown that NK cells become activated in patients undergoing acute rejection or CMV infection (4, 5). Collaborative research with the Doherty Institute has demonstrated a link between immune cells that control CMV and the later development of CLAD (6). We now wish to extend this biomarker approach with a new prospective cohort of LTx patients who will provide blood and BAL samples to allow further interrogation of immune and infectious links to allograft dysfunction following LTx.

Identifying, CLAD at an early stage, may allow therapeutic interventions to interrupt the progression to irreversible damage to the transplanted lung. We propose to analyse in both a cross-sectional and longitudinal study whether a series of biomarkers can predict for episodes of acute rejection or the development of CLAD in a cohort of patients undergoing LTx at the Alfred Hospital.

36. HYPOTHESIS

The identification of biomarkers of immune function and infection in the blood and lung will predict episodes of acute rejection and the onset of chronic lung allograft dysfunction (CLAD) following LTx.

37. BROAD PROJECT AIMS

4.1. To establish the immune response in the serum and broncholaveolar lavage (BAL) as measured cross-sectionally and longitudinally during the three year post-LTx and the association with later episodes of acute rejection or the subsequent development of CLAD.

4.2. To determine whether episodes of viral, fungal or bacterial infection in the LTx recipient trigger further increases in the secretion of these biomarkers and thus act as an inflammatory stimulus.

38. BACKGROUND

5.1 The immune response to lung transplantation

Successful outcomes following LTx depend on our ability to pharmacologically manipulate the immune system to prevent rejection of the lung allograft. The antigens involved in graft rejection are encoded by the major histocompatability complex (MHC). This group of highly polymorphic genes defines the human leukocyte antigens (HLA) which are expressed on virtually all cell surfaces. The polymorphic nature of these allopeptides defines the genetic signature of each individual and allows the host immune system to differentiate between self and foreign peptides. The majority of transplanted lung allografts are MHC-mismatched to the recipient. As such, the transplanted lung will be seen as foreign to the recipient's immune system. This leads to activation of immune pathways that if left unchecked would lead to rejection and destruction of the newly transplanted lung. We are interested in whether structural differences between transplant recipients and their donors, termed eplets, may drive the acute cellular and humoral rejection responses following LTx.

5.2 Chronic Lung Allograft Dysfunction

LTx chronic rejection has been described pathologically as Obliterative Bronchiolitis (OB) and physiologically as airflow limitation, in the form of the Bronchiolitis Obliterans Syndrome (BOS). However, increasingly other phenotypes of chronic rejection have been recognized, in particular the Restrictive Allograft Syndrome (RAS), characterized by restrictive physiology, interstitial infiltrates on radiology, and interstitial fibrosis, acute fibrinoid organizing pneumonia (AFOP) and diffuse alveolar damage (DAD) on histology (REF). To encompass these important emerging variants and concepts of chronic dysfunction, the term Chronic Lung Allograft Dysfunction (CLAD) has recently been created. We have been interested to see whether structural differences between transplant recipients and their donors may drive immune responses that lead to the later development of CLAD. Historically, the degree of matching between a transplant recipient and donor has been determined by counting the number of mismatched HLA-A, B and DR antigens of the donor. HLA antigens have multiple epitopes that can be recognised by specific antibodies. Elucidation of the three-dimensional molecular structures and amino acid sequence differences between HLA antigens allows structural characterisation of HLA antigens, and what are termed eplets. We have recently demonstrated that HLA matching at the eplet level protects against CLAD (7).

5.3 Acute Rejection

Despite the use of maintenance immunosuppression, acute cellular rejection is a common problem post lung transplant with 34% of adult LTx recipients experiencing at least one episode during the first year

post-transplant. The lung allograft is particularly susceptible to alloreactivity with the incidence of acute rejection being higher following LTx compared to other solid organ transplants. Whilst the direct effects of acute rejection are usually controlled and reversed following high-dose intravenous steroid treatment, the indirect effects remain problematic, with episodes of acute rejection being strongly associated with the later development of chronic lung allograft dysfunction (CLAD). Given the need for invasive transbronchial biopsies to provide a histological diagnosis of acute cellular rejection, we have previously evaluated biomarkers in the blood and BAL and their association with acute rejection (2-5). Functional assays of T cell and B cell function in the blood may identify patients at higher risk of acute cellular rejection. Analysis of the BAL is very useful in providing mechanistic insights into the immunology of graft function and rejection following LTx. However, BAL findings seen in acute rejection must be interpreted in the context of those described in stable lung transplant recipients, and after taking into account the confounding nature of chronic airway infection.

5.4 Infection in immunosuppressed lung transplant recipients

Infection is commonly encountered following LTx and is largely related to the use of immunosuppressive medication. We have a long-standing interest in how opportunistic infections such as CMV (8) and Aspergillus (9) have both direct effects on the lung allograft, as well as indirect effects through an association with CLAD. The airways, historically thought to be sterile, are now known to harbor a microbiota that changes in its constituents depending upon the health status of an individual. This lung microbiome consists of bacteria, viruses and fungi, which are in intimate contact with cells in the lung providing tonic stimuli to our immune system. As is routine clinical practice, the current study will also include a detailed prospective analysis of infections, and associated risk factors in the cohort of LTx recipients. In particular, we will determine the impact and interaction of bacteria, viruses and fungi in the transplanted lung, in order to inform improved treatment and prevention of CLAD.

39. STUDY PROPOSAL

LTx saves lives, however the long-term viability of the allograft is limited by the immune response to it and the development of CLAD. The production of *de novo* anti-HLA alloreactive donor-specific antibodies (DSA) is deleterious to the graft and is associated with CLAD. However, the immune cell response following LTx and its relationship to the lung microbiome is poorly understood. We propose a thorough and integrated delineation of the immune response to LTx. We will obtain a comprehensive picture of the lower airway bacterial, viral and fungal communities in LTx recipients, and determine the implications of the lung microbiomeon inflammation, lung tissue remodelling pathways and allograft function. Following LTx, we will have defined immune and physiological biomarkers of rejection that will better inform the use of appropriately focused immunosuppressive therapies with a

view to reducing CLAD and prolonging survival following LTx. Insights gained will extend beyond LTx to all types of solid organ transplantation.

40. RESEARCH PLAN

Specific Aim #1: Defining HLA eplet mismatch scores

Rationale: The humoral antibody-antigen immune response describes the interaction between anti-HLA DSA with the cognate non-self HLA molecule expressed within the lung allograft. HLA genes are highly polymorphic with over 10,000 HLA Class I and II alleles. Anti-HLA antibodies recognise specific exposed regions of the HLA antigen that consist of chains of amino acid sequence located within the complement determining regions, termed eplets.. Approximately 600 HLA Class I and II eplets have been described by Duquesnoy *et al.* (10) using the theoretical computer algorithm HLAMatchmaker. Functional eplets specifically recognise HLA antibodies and are exposed within the binding groove demonstrating antigenicity, i.e. reactivity with antibody, and immunogenicity, i.e. ability to elicit immune response. LTx recipients are transplanted with HLA-mismatched donor lungs. HLA typing will be performed by Luminex sequence-specific oligonucleotide or sequenced-based typing for HLA-A, -B, -C, -DR and -DQ (Victorian Transplantation and Immunogenetics Service). The HLAMatchmaker 500 pair (ABC and DRDQ eplet) program will be used as a research tool to assess eplet matching for all LTx (http://www.hlamatchmaker.net/). The calculated linear score of structural diversity for each LTx will be used for the subsequent studies looking at the impact of structural incompatibility on immune pathways and allograft dysfunction.

Specific Aim #2: Immune response in high eplet mismatch lung transplant recipients

x) De novo DSA production

Rationale: Anti-HLA DSA may be present at the time of LTx (sensitised recipient) or develop *de novo* following LTx, and may be specific to HLA-class I and/or HLA Class II. The alloreactivity of DSA is determined by whether they are bound within the lung allograft, and whether they amplify an immune response. The C1q assay is a modification of the standard Luminex assay that distinguishes complement-fixing (and therefore injurious) from non-complement-fixing DSA. The clinical utility of the C1q assay has been demonstrated in renal transplantation, and similar studies are awaited in LTx. DSA can also be alloreactive via complement-independent pathways. NK cells and macrophages can be directly activated via the low-affinity Fc receptor resulting in antibody-independent cell-mediated cytotoxicity. Aim #2 will integrate DSA results with eplet mismatch scores and other immune markers to provide a comprehensive description of Antibody Mediated Rejection (AMR).

xi) B-cell specificity, phenotype and function

Rationale: B cells can be alloreactive, either as precursors (naïve or memory B cells) of DSA-producing plasma cells or as antigen-presenting cells to allospecific T-cells. HLA tetramers provide an opportunity to track antigen-specific B cell responses longitudinally from the time of LTx. In LTx, the dynamics

and role of alloreactive B cells in producing anti-HLA DSA and graft dysfunction has not been described.

B cell subtypes will be phenotyped from the blood of LTx recipients by flow cytometry in the lab of Prof Function of immune cells will be assessed using a stimulated intracellular cytokine stain. The dynamics of B and T cell biology will be tracked longitudinally over the first 12 months, and will be correlated with eplet mismatch scores and *de novo* DSA production (assessed at 3 and 12 months post-transplant).

xii) Antibody-dependent NK cell cytotoxicity

Rationale: The complement-independent mechanisms that lead to AMR are poorly understood, although recent work suggests that NK cells may be important effectors of Antibody-Dependent Cell-Mediated Cytoxicity (ADCC). We have previously shown that NK cells are activated in LTx recipients and contribute to DSA-mediated allograft injury (5). Molecular signatures from allograft biopsies support the alloreactive potential of NK cells. We aim to use an *in vitro* NK-cell ADCC assay to analyse the role of DSAs in promoting NK cell activation. NK cells will be identified by flow cytometry (CD3-CD56+ lymphocytes) in the blood LTx patients at 3 and 12 months post-transplant.

Specific Aim #3: Determine the immunological profiles of the lower airways of lung transplant recipients

We will characterise the immunological environment of the lower airways of LTx recipients at each timepoint (i.e. 2 weeks, 3, 6, 9, 12 and 18 months) by RNAseq of immune cells isolated from the lavage fluid. We will perform Mass Spectrometry of the lavage fluid to characterise the metabolome, lipidome and proteome. Bioinformatic integration of these datasets (see Aim 5) will allow us to gain deep insight into the immunological profiles of the patient groups.

Specific Aim #4: Identification of the bacterial, fungal and viral communities in lung transplant recipients

Bacterial and fungal community identification: Fungal and bacterial DNA will be amplified and sequenced. The virome will be characterised, using two experimental approaches in order to ensure we capture a complete and informative dataset. First, PCR amplification will be used to analyse the total set of BAL samples informing on the carriage of a specific set of RNA and DNA viruses, including a panel of anelloviruses which we hypothesize will be highly relevant in the lung transplant setting. In a second step, we will refine the patient pool for analysis based upon the clinical, immunological, bacterial, fungal and viral PCR results and perform metagenomic sequencing providing us with in-depth unbiased data on viral carriage.

Specific Aim #5: Bioinformatics and integration of microbial, immunological and clinical datasets

Network-based approaches have emerged as a powerful way to study microbial systems and analyse high-dimensional datasets. Using the bacterial, fungal and viral sequencing datasets, we will construct and combine ecological networks using dissimilarity and correlation-based methods. This will firstly allow us to determine the extent to which bacterial, fungal and viral communities are connected by causal relationships, and in particular, we can identify "key-stone" species which would be predicted to play a major role in community dynamics and consequently the immunological and health status of the transplant recipient.

Specific Aim #6: Applying microarray to describe the molecular phenotype of lung allografts
The current standard of biopsy-based assessment of lung transplants is the ISHLT histology classification applied to transbronchial biopsies. This assessment is based largely on empirical classifications and expert opinions, and provides little insight into the pathophysiological and immunological drivers of CLAD. Microarray analysis establishes diagnostic patterns of abnormality (and normality), but as yet is not been rigorously applied in the LTx setting.

We will utilise Microarray Analysis at 3 and 12 months post-transplant, scores provided by the Molecular Microscope Diagnostic System used to guide diagnosis of T cell mediated rejection and AMR will be applied to the lung allograft biopsies provided. These Microarray results will be correlated with eplet mismatch scores and immune markers and CLAD.

SUB-STUDY: Single Lung Transplant (SLTx) Recipients
From January 2021, the study will continue to recruit only participants who receive a single lung transplant. We will perform Specific Aims 3-5 above, on the lavage fluid from both the transplanted lobe and the native lobe. The datasets generated from the different lobes will be compared in order to ascertain whether the immunological and microbiological characteristics of the transplanted lobe develops independently, or in parallel, with the native lobes. This analysis will inform whether the local microenvironmental changes within individual lobes influence the health trajectory of transplant recipients.

41. METHODOLOGY

8.1 Patient Recruitment:

All adult patients undergoing lung transplantation, and whose long-term follow-up will be at the Alfred Hospital will be invited to participate in the study. Patients would be enrolled prior to transplantation.

8.2 Sample Collection:

8.2.1 BAL research samples will be collected throughout the project at time points and quantities described here.

At Bronchoscopies:

g) As part of routine post-transplant management lung transplant recipients attend for surveillance bronchoscopy at 1 day, 2 and 6 weeks and 3, 6, 9, 12 and 18 months post-transplant, at which point BAL samples are collected.
h) On occasions of clinical concern, additional bronchoscopies may be scheduled as part of patient care. At these clinical relevant bronchoscopies, BAL samples are also collected.

30mls of BAL will also be collected from both the transplanted lung and the native lung of participants who receive a single lung transplant

8.3 Participant Involvement:

All participants will be followed for a total of 3 years following lung transplant.

Lung transplant recipients enrolled in the study would not need to make any additional trips to the Alfred hospital nor undertake any additional investigations. Collection of research related samples at bronchoscopies will coincide with routine biopsy, blood and BAL collections and will not require additional procedures for the participants.

8.4 Sample Processing:

At bronchoscopy, blood and BAL samples will be collected from the transplanted lung according to standard clinical protocols and forwarded to microbiology for routine diagnostic assessments. These microbiological results will be analysed as an outcome variable in the current study.

Remaining BAL samples from both lungs will be processed and centrifuged at the Alfred hospital to BAL supernatant samples for biomarker analysis. These samples will then be transported to our collaborators for analysis.

42. INCLUSION/EXCLUSION CRITERIA

Approximately 150 patients, including 10-15 single lung transplant recipients, will be enrolled at The Alfred Hospital

Inclusion Criteria

- Adults ≥18 years of age
- Lung Transplant Recipients
- Provide Written Consent

Exclusion Criteria

- Long Term Follow-Up not planned for The Alfred Hospital

43. ANALYSIS:

Descriptive statistics will be used to determine the baseline and clinical characteristics of the subjects in the study. Univariate analysis of the biomarkers and their association with the clinical end-points of acute rejection and CLAD will be performed using the Chi-square test for equal proportion, Student's t test for normally distributed continuous variables and the Mann-Whitney U test for non-normally distributed variables. Time to acute rejection and CLAD will be analyzed using univariate and multivariate Cox proportional hazards regression, adjusting for potential confounding factors and reported using the Kaplan Meier curves. All analysis will be performed using SAS version 9.2 (SAS Institute Inc., Cary, NC, USA). A two-sided p-value of 0.05 will be considered to be statistically significant.

44. REFERENCES

31. Paraskeva M et al. (2013) Acute fibrinoid organizing pneumonia after lung transplantation. *Am J Respir Crit Care Med* 187, 1360-1368.

32. Westall G et al. (2017) Activin Biology after Lung Transplantation. *Transplant Direct* 11; 3:e159.

33. Snell G et al. (2015) The potential role of activin and follistatin in lung transplant dysfunction. *Expert Rev Respir Med* 9:697

34. Meehan AC et al. (2013) The impact of commonly used transplant immunosuppressive drugs on human NK cell function is dependent upon stimulation conditions. *PLoS One* 8:e56042

35. Meehan Ac et al. (2010) Natural killer cell activation in the lung allograft early posttransplantation. *Transplantation* 89:756

36. Sullivan LC et al (2015) The presence of HLA-restricted, CMV-specific CD8+ T cells in the blood of human lung transplant recipients correlates with chronic allograft rejection. *PLos One* 10:e135972

37. Walton D et al (2016) HLA matching at the eplet level protects against chronic lung allograft dysfunction. *Am J Transpl* 16:2695

38. Paraskeva M et al. (2011) CMV replication within the lung allograft is associated with bronchiolitis obliterans syndrome. *Am J Transpl* 11: 2190.

39. Jeong W et al (2017). Clinical effectiveness of early posaconazole suspension pre-emptive therapy in lung transplant recipients. Antimicrob Chemother epub ahead of print

40. Duquesnoy RJ (2016) Reflections on HLA epitope based matching for transplantation. Front Immunol 7:469
